# Metabolic implications of axonal demyelination and its consequences for synchronized network activity: An *in silico* and *in vitro* study

**DOI:** 10.1177/0271678X231170746

**Published:** 2023-04-26

**Authors:** Zoltan Gerevich, Richard Kovács, Agustin Liotta, Luisa A Hasam-Henderson, Ludwig Weh, Iwona Wallach, Nikolaus Berndt

**Affiliations:** 1 Institute of Neurophysiology, Charité – Universitätsmedizin Berlin, corporate member of Freie Universität Berlin and Humboldt-Universität zu Berlin, Berlin, Germany; 2 Department of Anesthesiology and Intensive Care, Charité – Universitätsmedizin Berlin, corporate member of Freie Universität Berlin and Humboldt-Universität zu Berlin, Berlin, Germany; 3 Institute of Computer-assisted Cardiovascular Medicine, Deutsches Herzzentrum der Charité (DHZC), Berlin, Germany; 4 Institute of Biochemistry, Charité – Universitätsmedizin Berlin, corporate member of Freie Universität Berlin and Humboldt-Universität zu Berlin, Berlin, Germany; 5 Charité – Universitätsmedizin Berlin, corporate member of Freie Universität Berlin and Humboldt-Universität zu Berlin, Berlin, Germany

**Keywords:** Demyelination, action potential propagation, energy consumption, desynchronization of network activity, mathematical modeling

## Abstract

Myelination enhances the conduction velocity of action potentials (AP) and increases energy efficiency. Thick myelin sheaths are typically found on large-distance axonal connections or in fast-spiking interneurons, which are critical for synchronizing neuronal networks during gamma-band oscillations. Loss of myelin sheath is associated with multiple alterations in axonal architecture leading to impaired AP propagation. While numerous studies are devoted to the effects of demyelination on conduction velocity, the metabolic effects and the consequences for network synchronization have not been investigated. Here we present a unifying computational model for electrophysiology and metabolism of the myelinated axon. The computational model suggested that demyelination not only decreases the AP speed but AP propagation in demyelinated axons requires compensatory processes like mitochondrial mass increase and a switch from saltatory to continuous propagation to rescue axon functionality at the cost of reduced AP propagation speed and increased energy expenditure. Indeed, these predictions were proven to be true in a culture model of demyelination where the pharmacologically-induced loss of myelin was associated with increased oxygen consumption rates, and a significant broadening of bandwidth as well as a decrease in the power of gamma oscillations.

## Introduction

Proper brain function relies on the ability to process and transfer information in form of action potentials (AP) at a reasonable metabolic cost. Electrical insulation of the axons increases both the energy efficiency and conduction velocity of APs.^1^ Oligodendrocytes provide an insulating ensheathment of axon fibers forming myelinated internodal segments with periodic unmyelinated gaps of high Na_v_ channel density, known as nodes of Ranvier (NOR). The NORs are framed by paranodal myelin binding sites and juxtaparanodal regions with high potassium channel density^2–4^ allowing for precise and fast AP conduction.^5,6^

A respiratory quotient close to 1 indicates an almost exclusively carbohydrate-based oxidative metabolism in the brain. More than 90% of the glucose is used to generate ATP via oxidative phosphorylation and about 2/3 of the total cellular ATP production (including aerobic glycolysis) is spent on the membranous ion pump Na-K-ATPase, which builds up the ion gradients required for the generation of APs.^7^ As transmembrane ion fluxes and transport mechanisms are restricted to NOR, myelinated fibers work more energy-efficient than completely unmyelinated axons, which is particularly important in the case of high-frequency AP firing.^8,9^

In the cerebral cortex, parvalbumin-positive (PV^+^) interneurons are the most frequently myelinated neurons contributing up to 80% of the myelin in the hippocampus.^10–12^ They fire narrow APs at extraordinarily high frequency and the metabolic cost of high-frequency AP firing is mirrored in the mitochondrial mass and intensive oxidative metabolism of PV^+^ interneurons.^13^ Similar to the number and size of mitochondria, myelination of interneurons is plastic and correlates with the activity of the particular cell type.^11^

PV^+^ interneurons play a key role in the synchronization of network activity in the gamma (30–80 Hz) and higher frequency ranges by delivering rhythmic perisomatic inhibition onto pyramidal cells.^14,15^ Gamma oscillations are proposed to promote neural communication by synchronizing local neuronal ensembles or distant brain regions and are implicated in primary sensory processing, attention, and short- and long-term memory.^16,17^ Network synchronization in the millisecond range requires fast and reliable AP propagation and a precisely timed GABA release from the heavily ramified PV^+^ axons^18^ with a total length of up to 53 mm and lateral distribution of 780–1740 μm.^19^ Consequently, any disturbance of the myelination would also affect network synchronization. Indeed, myelin defects and disturbances in gamma activity have been described in schizophrenia patients^20^ and animal models of the disease.^21^

Demyelination disease describes the loss of myelin sheath and is associated with multiple alterations in axonal architecture. Early changes include the widening of the NOR, characterized by the insertion of an unmyelinated membrane, resulting in splitting the sodium channel enriched node into two heminodes separated by an ion channel-free membrane.^22^ As the demyelination process progresses, retraction of myelin from the paranodal and juxtaparanodal structure exposes potassium-channel rich juxtaparanodal stretches to the extracellular space.^22^ Finally, the demyelinated axon segment can reach lengths up to 200 µm^22^ thereby disrupting reliable signal transmission.^23,24^ Demyelination can lead to slowing or even blocking of AP propagation, axonal damage, and severe neurological impairment.^20,25–27^ Loss of myelin may also propose a major threat to the axonal energy balance as loss of insulation would increase ATP demand for the maintenance of ion gradients. Consequently, to cope with myelin damage, an increase in mitochondrial density has been suggested as an adaptation mechanism during partial myelin loss and complete demyelination.^28,29^

To regain functionality and prevent conduction block, the demyelinated membrane can be rendered excitable by the insertion of voltage-gated ion channels.^30,31^ It remains a question, though, if such an adaptation would be energetically maladaptive on a tissue-wide scale. Conduction block and the effect of nodal disruption and demyelination on AP propagation have been studied extensively (see Coggan et al.^25^ and references within) whereas the metabolic implications of disrupted myelin architecture have not been addressed so far.

Here, we present a mathematical model of myelinated axon coupling electrophysiological processes occurring during AP propagation with a kinetic model of neuronal energy metabolism.^32^ We explore the model to study the effects of progressive demyelination on AP propagation and energy efficiency. Our model represents relevant ion and metabolite concentrations (such as sodium, potassium, ATP, glucose, lactate, etc.), as independent variables and thus allows assessing their changes during different phases of demyelination disease. We tested the predictions of this model on neuronal signal processing by using a pharmacological demyelination paradigm in organotypic brain slice cultures and measured 1) the oxygen consumption rate and 2) the parameters of cholinergically induced gamma oscillations. Since most myelinated axons in the cortex belong to the PV^+^ interneurons generating gamma oscillations and axons account for only a small proportion of the total energy demand of the tissue, the parameters of gamma oscillations represent a sensitive measure to validate our model. Already small changes in gamma oscillations might indicate that the axons PV^+^ interneurons are affected. We show that demyelination of slice cultures increases oxygen consumption rates and disrupts the synchrony of gamma oscillations without an immediate effect on the viability of the neurons.

## Materials and methods

### Model description

**Figure 1.**
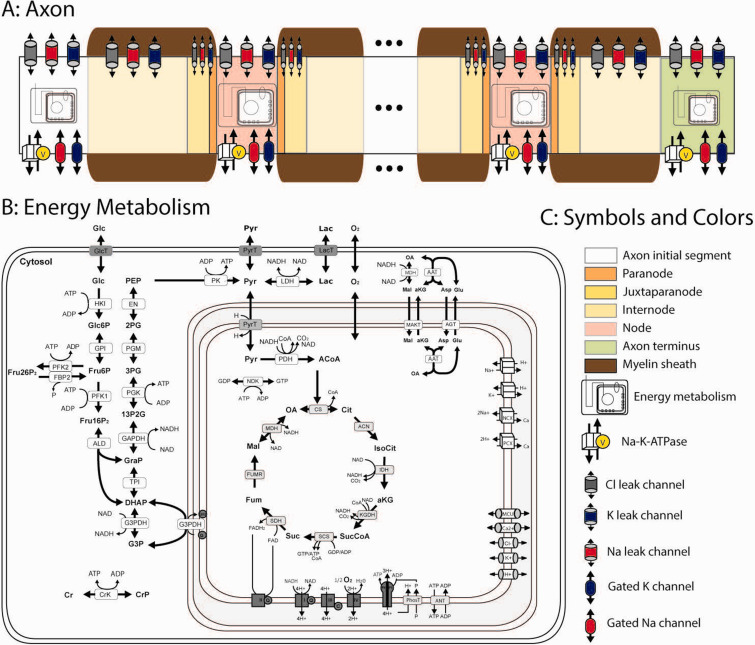
(a) Axon structure as used for simulations (not to scale). Action potentials are elicited at the axon initial segment (AIS) and pass through a sequence of alternating, electrically passive, myelinated axon stretches and nodes of Ranvier (NOR) as electrically active segments before reaching the axon terminus. AIS and axon terminus include Hodgkin-Huxley standard ion conductivities; the NOR segment is represented as a 1 μm Na_V_-rich membrane flanked by 40 μm proximal and distal K_V_-rich juxtaparanodal membrane. All electrically active segments show Na-K-ATPase activity and leak conductivity for Na, K, and Cl ions. Internodes are channel and ATPase-free. (b) Reactions and transport processes as included in the metabolic model. The model comprises the following subsystems: conversion of glucose to pyruvate/lactate in glycolysis; shuttles of electrons (NAD-bound hydrogen) between the cytosol and the mitochondrial matrix combining the activities of the malate/α-ketoglutarate carrier (MAC); the mitochondrial aspartate aminotransferase (AAT); the exchange of glutamate (Glu) and aspartate (Asp) by the aspartate/glutamate carrier (AGC); the glycerol-3-phosphate (G3P) shuttle comprising mitochondrial and cytosolic G3P dehydrogenases (G3PDH); the tricarboxylic acid cycle starting with the formation of citrate (Cit) from oxaloacetate (OA) and acetyl-CoA (ACoA), and replenishing OA in a circular chain of reactions yielding electrons in terms of ubiquinol (QH_2_) and NADH, and two molecules CO_2_; and the respiratory chain composed of four complexes of which complexes I, III, and IV function as proton pumps. The resulting proton gradient and mitochondrial membrane potential determine the rate of proton-assisted ion transport of Na^+^, K^+^, Ca^2+^, and phosphate (P) across the inner mitochondrial membrane, the rate of the adenine nucleotide exchanger (NE) exchanging mitochondrial ATP against cytosolic ADP, and the rate of F0F1-ATPase using three protons per generation per ATP. The mitochondrial membrane potential is also inﬂuenced by the exchange of cations and anions across the inner mitochondrial membrane through ion channels. Lactate is exchanged with the external space by the monocarboxylate transporter (MCT2). Within the cell, it is reversibly converted into pyruvate by lactate dehydrogenase (LDH) and (c) Symbols and colors used in (a).

The model describes the movement of sodium, potassium, chloride, and calcium ions across the axonal plasma membrane, the generation and propagation of APs along the axon, and the axonal energy metabolism. The simplified axon is represented as a hollow cylinder divided into segments with homogenous metabolite and ion concentrations. These segments differ in their endowment of metabolic enzymes and electrical properties (channel densities, membrane capacity, and degree of myelination) depending on the segment type. Segment types comprise the axon initial segment (AIS), the internode, the juxtaparanode, the paranode, the NOR, and the axon terminus (AT). In each segment, the model discriminates between extracellular space, axonal lumen, and mitochondrial space. The axonal model structure is depicted in Figure 1(a).

#### Electrophysiological sub-model

The electrophysiological sub-model describes the dynamics of sodium, potassium, chloride, and calcium ion fluxes as well as the membrane potential. Changes in ion concentrations in the extra-axonal space, the axonal lumen, and the mitochondria are the result of the different elementary processes considered in this model:

- Diffusive ion movement within the extra-axonal space according to Fick’s first law;
- Electro-diffusive ion movement within the axonal lumen;
- Electro-diffusive movement of ions through voltage-dependent and voltage-independent ion channels in the axonal plasma membrane;
- Electro-diffusive movement of ions through voltage-dependent and voltage-independent ion channels in the mitochondrial membrane;
- Active (ATP-dependent) transport of ions through the axonal plasma membrane;
- Active (proton-dependent) transport of ions through the mitochondrial membrane.

Ion movements across the membrane and membrane potential changes are modeled via the Goldman-Hodgkin-Katz equation.^33^ Changes in the axonal plasma membrane potential in any segment result from ion movement across the membrane of that segment and electrical coupling between adjacent axonal segments. Resembling the approach used in^34^ to model AP propagation with a difference-differential equation, the model considers the membrane potential a quasi-diffusible variable, i.e. propagation velocity is defined by the axoplasmic resistivity value. The electrophysiological reaction scheme for a representative axon segment is depicted in Figure 1(a).

#### Effect of myelin and modeling of demyelination

Myelin decreases the effective capacitance of the axonal membrane. Typical myelin shielding along the axon plasma membrane reduces membrane capacitance to about 0.0015 times the unmyelinated value.^5^ Depicting the myelinated axon as concentric cylinders, the membrane capacitance can be described by $c_{\mathrm{axon}}∼\log(r_{1}/r_{2})$ , with $r_{1}$ being the inner axon and $r_{2}$ being the outer myelin radius. The resulting dependence on membranous capacitance on myelination is as described in Koles and Rasminsky.^35^ Additionally, myelin acts as an insulator decreasing the effective permeability of ion channels in the myelinated membrane stretches. Demyelination exposes these ion channels thereby increasing their conductance. This initiates passive countercurrents along the demyelinated membrane stretches resulting in the dampening of APs. Thus, the dampening of APs is not introduced via a dampening term in the propagation equation, but the consequence of the channel exposure during demyelination. All model equations are given in the Supplement.

#### Metabolic sub-model

The metabolic sub-model comprises the glycolytic pathway, the malate-aspartate shuttle, the glycerol-3-phosphate shuttle, the mitochondrial tricarboxylic acid cycle, the respiratory chain, the mitochondrial electrophysiology, and the oxidative phosphorylation for ATP generation. Regulation of metabolic enzymes and transporters considered in this model comprises substrate affinities (K_m_ values), allosteric regulation via activation (K_a_ values), and inhibitions (K_i_ values). The model is based on the design of previously published models of neuronal energy metabolism.^32,36–38^ The numerical values for all kinetic parameters were taken from reported kinetic studies of the isolated enzyme. The Vmax values represent fit values to obtain correct flux rates and metabolite concentrations. Metabolite concentration, fluxes, and dynamic NADH transients as well as original literature for collecting these data are given in Berndt et al, 2015.^32^ The metabolic reaction scheme is depicted in Figure 1(b). All model equations for the metabolic part are also given in the Supplement.

#### Axonal structure

The axon is composed of various segments differing in their electrophysiological properties (Figure 1(a)). The AP is generated at the AIS. It is composed of an unmyelinated membrane containing voltage-gated sodium and potassium channels, basal sodium, potassium, and chloride channels, and ligand-gated sodium channels. The AIS contains high amounts of Na-K-ATPase and the whole energy-producing machinery (i.e. glycolytic protein and mitochondria). The internodal segments are myelinated membrane stretches. As ion channels are almost absent, only basal sodium, potassium, and chloride permeabilities are considered. The juxtaparanode segments are myelinated segments that contain large clusters of voltage-dependent potassium channels in addition to basal ion permeabilities. The paranodal segments are the ones tightly binding the myelin to the plasma membrane. They contain basal ion permeability and are treated as myelinated segments. The NOR represent short unmyelinated gaps within the axon membrane that are characterized by a very high density of fast-gated sodium channels. They also contain NA-K-ATPase and the whole energy-producing machinery. The AT is the unmyelinated end of the axon where the synapse is located. It contains voltage-gated sodium and potassium channels as well as basal sodium, potassium, and chloride channels. It also contains NA-K-ATPase and the whole energy-producing machinery. The modeled axon consists of the AIS followed by six internodal stretches interrupted by five NOR and a final AT. All model equations and parameters are given in the Supplement.

#### Coupling energy metabolism to neuronal excitation

Most axonal mitochondria are located in stationary pools in the internode and juxtaparanode. Increased electrical activity will slow or arrest their movement near the NOR, probably in response to higher energy demand during AP firing.^39,40^ To cope with increased energy demand, mitochondria grow in size and move to the required axon sites faster.^41^ Integrating these findings, we assume metabolic activity only in the AIS, NOR, and AT segments of our model, while internode, paranode, and juxtaparanode segments remain metabolically passive. Coupling proceeds by ATP hydrolyzation by the ATP-dependent pumps in the active segments. ADP and phosphate are then used for ATP synthesis by glycolysis or oxidative phosphorylation. All model simulations were performed using MATLAB, Release R2022a, The MathWorks Inc., Natick, Massachusetts, United States.

### Preparation of organotypic hippocampal slice cultures and demyelination protocol

Organotypic hippocampal slice cultures were prepared from 6–7 postnatal-day-old wild-type or transgenic Wistar rats expressing venus yellow fluorescent protein (YFP) on the vesicular GABA transporter (VGAT) promoter^42^ of either sex following the guidelines of the European Union (Directive 2010/63/EU) and the institutional guidelines, approved by the Berlin Animal Ethics Committee (Landesamt für Gesundheit und Soziales Berlin, G0437/12) as previously described.^15^ The experiments have been reported following the ARRIVE guidelines.^43^ Briefly, hippocampi were extracted and cut into 400 μm slices perpendicular to the dorsoventral axis with a McIllvain Tissue Chopper. Slices were seeded on PTFE membranes (Millicell-CM, Millipore) and maintained in six-well plates filled with 1 mL culture medium (50% MEM, 25% HBSS, 25% Horse Serum, and 1 mM L-glutamine, pH set to 7.3) in a humidified CO_2_ incubator (5% CO_2_) and left to recover for 7 days *in vitro* (DIV). For demyelination, lysophosphatidylcholine (LPC, Sigma) was solved in ethanol (EtOH, 4 g/100 mL) and applied at a final concentration of 0.5 mg/mL to the medium after 7 DIV for 15–17 h at 37°C as described previously.^44^ In the solvent control experiments, slice cultures were incubated only in ethanol (9.48 µg/L). Cultures were randomly assigned to the different treatment groups. After incubation, the LPC and EtOH-containing media were removed and replaced with a fresh medium.

### Oxygen electrode recordings

Slice cultures were transferred to interface-type recording chambers and perfused with warm (36.4°C) and carbogenated artificial cerebrospinal fluid (aCSF) containing (in mM): NaCl 129, KCl 3, NaH_2_PO_4_ 1.25, MgSO_4_ 1.8, CaCl_2_ 1.6, NaHCO_3_ 26, and glucose 10 at a flow rate of 2 mL min^−1^. Oxygen gradients were recorded with Clark-style oxygen sensors (tip: 10 µm; Unisense, Aarhus, Denmark) as previously described.^45^ Oxygen electrodes were polarized for >12 h and two point calibrated in aCSF gassed with 50% and 95% O_2_ before each recording session. For depth profiles, the pO_2_-electrode was fixed to a mechanical manipulator, positioned at the stratum pyramidale of area CA3, and moved vertically through the slice in steps of 20 µm until additional steps no longer reduced pO_2_.

### Electrophysiology

Local field potentials were recorded from the stratum pyramidale of CA3 with glass pipettes filled with aCSF (<4 MΩ) using an EXB-EXT-02B NPI Electronic amplifier (Norbert Polder Instruments, Germany), high-pass filtered at 0.1 Hz, low-pass filtered at 1 kHz, and sampled at 5 kHz by a digitizer CED Micro1401-2 with an ADC12 extension (Cambridge Electronic Design Limited, Cambridge, UK).^15^ Gamma oscillations were induced by the application of carbachol (CCh, 20 μM) to the perfusion after 10 min of baseline.

### Analysis and statistics of gamma oscillations

Experiments were designed to generate groups of equal size. Slices were randomized into LPC-treated, solvent-treated (EtOH), or control groups before treatment. Group size selection was planned by the program G*Power3.^46^ Only stable and drug-induced gamma oscillations were analyzed. For blinding, operators and analysts were different persons. Power spectra were calculated every 2 min with a 120-s window throughout the recording. Based on these power spectra, peak power, peak frequency, and half bandwidth of the oscillations were determined off-line by using a custom-made script for the Spike2 software (version 7.10, Cambridge Electronic Design, Cambridge, UK).^15,47^ The mean values of a 20 min period immediately after the application of CCh (0–20 min) and 100 min later (100–120) were calculated and compared to each other. The D'Agostino-Pearson normality test was used to test the Gaussian distribution of the data. Absolute power values displayed a log-normal distribution and were therefore presented as a geometric mean with a 95% confidence interval. All other data were presented as mean ± SD. In graphs, lines represent geometric or arithmetic means of log-normally or normally distributed data, respectively. Statistical analysis of log-normally distributed power values was performed using the Kruskal-Wallis test with Dunn’s post hoc multiple comparisons. The peak frequency and bandwidth of the oscillations were compared by using one-way ANOVA and Tukey’s post hoc test. The significance level was set at P < 0.05. The sample size “n” refers to the number of slices that were taken from the brains of the indicated number of animals referred to as “N”. More slices from each animal were used for experiments but not more than one slice per hemisphere for each treatment group.

### Immunohistochemistry

Organotypic hippocampal slice cultures were fixed with paraformaldehyde 4%/sucrose 4% in phosphate-buffered saline (PBS) 0.1 M overnight at 4°C. After fixation, slice cultures were washed with PBS and detached from the insert membranes. Slices were processed free-floating in wells and rinsed with PBS between steps. Slices were exposed overnight to a solution containing 10% normal goat serum (GS) and Triton X 0.5%. Slices were incubated for three nights with the respective primary antibodies (anti-Myelin Basic Protein, MBP, 1:1000, ThermoFisher; anti-parvalbumin, PV, 1:1000, Millipore) in 0.3% Triton X/5% GS/PBS. This was followed by an overnight incubation (0.1% Triton X/3% GS/PBS) with a goat anti-rabbit Alexa488 or goat anti-mouse Cy3 secondary antibody (1:100; Millipore). Slices were mounted on gold-coated slides and coverslipped with Fluoromount-G Mounting Medium (SouthernBiotech).

### Image acquisition

An overview of the hippocampal formation was obtained for each slice with an inverted confocal microscope (Leica DMI 6000) with a 40× oil immersion objective. For each slice, 20 to 30 z-stacks were obtained with a distance of 1.2 µm. Reconstructions of individual NG2 or MBP/PV co-labeled slices were carried out with a Leica SP5 confocal microscope. Image processing was performed using ImageJ (Fiji release 1.51; Wayne Rasband, NIH, USA). To reveal the myelination of fast-spiking interneurons in living slice cultures we used a combination of third harmonic generation (THG) and multiphoton fluorescence microscopy. We took advantage of the fact that the oxidation-sensitive fluorescent probe, chloromethyl-2,7-dichlorodihydrofluorescein (CM-H_2_DCF) is selectively oxidized and accumulates in fast-spiking interneurons.^13^ Slice cultures were incubated for 20 min with the membrane-permeable diacetate-form of the probe (10 µM) in the incubator and moved to the perfused submerged recording chamber in a NIKON A1R multiphoton microscope (25× objective, N.A. 1.1, Nikon, Shinagawa, Tokyo, Japan) at the AMBIO Life Cell Imaging Core Facility (AMBIO.charite.de). THG and DCF fluorescence excitation was achieved at 1180 nm and 1040 nm respectively with a pulsed Ti:Sa Laser (InSight DeepSee, Spectra-Physics, Santa Clara, CA, US). DCF fluorescence emission and backward scattered THG signal were recorded at 400–450 nm and 500–530 nm channels, respectively.

## Results

We defined a ‘generic’ axon by the following key functional and metabolic features: (1) AP amplitude is ∼100 mV. (2) AP propagation speed is ∼3 m/s. (3) AP amplitude is preserved at the NOR during propagation (see Supplementary Figure S1). (4) Ten percent of glucose is converted into lactate. (5) The relative shares of the proton leak, the pumping of potassium ions, and ATP synthesis (including F0F1-ATPase and phosphate uptake) in the utilization of the proton gradient amount to 24%, 16%, and 60%, respectively. (6) The mitochondrial transmembrane potential has a value of −140 mV. (6) ATP content is ∼3 mM. The metabolic model is based on Berndt et al.^32^ All model equations are given in the Supplement.

To investigate the effect of demyelination disease on AP propagation and metabolism we simulated the effect of node widening, paranodal demyelination, and segmental demyelination.

### Node widening

**Figure 2.**
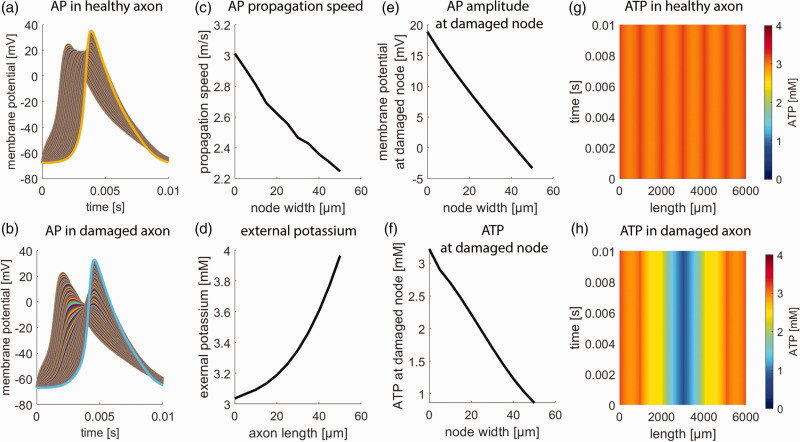
Effects of node widening. (a) Action potential (AP) propagation over time in a healthy axon and (b) in an axon with 50 µm node widening at the 3rd NOR. The different curves give the membrane potential of each axon segment. The decreased AP amplitude results from the widening of the 3rd NOR. (c) AP propagation speed in dependence on node width. Propagation speed decreases in dependence on node widening at the 3rd NOR. (d) Extra-axonal potassium concentration around the damaged node in dependence on node widening. Increased node width leads to increasingly aberrant potassium concentrations around the damaged node. (e) Maximal axonal membrane potential during AP at the damaged node in dependence on node width. Increased node width decreases AP amplitude around the damaged node. (f) ATP concentration at the damaged node in dependence on node width. Increased node width leads to energy depletion at the damaged node. (g) Axonal ATP concentration in a healthy axon. The x-axis depicts the position along the length of the axon, y-axis depicts time along an AP (same as the x-axis in panels A–F). ATP concentrations are around 3 mM throughout the whole axon. Constancy over time indicated that perturbance by a single AP can be buffered by available ATP and (h) Axonal ATP concentration in an axon with 50 µm node widening at the 3rd NOR. ATP depletion centers around the damaged node but spreads through the axon.

First, node widening was simulated by the insertion of an unmyelinated membrane at the third NOR. This leads to a splitting of the node into two heminodes. We varied the insertion width between 0 µm (healthy axon) and 50 µm.^22^ Figure 2 shows the effects of node widening on axonal AP characteristics. Increasing node width decreases the AP propagation velocity from ∼3 m/s to about ∼2.2 m/s in comparison to the AP propagation in a healthy axon (Figure 2(a) to (c)), and the AP amplitude around the damaged node decreases by more than 20 mV without a reduction in AP amplitude at the AT (Figure 2(a), (b) and (d)). This is accompanied by a severe energy depletion around the damaged node (Figure 2(g)) with ATP decreasing from ∼3 mM to about ∼1 mM. The energy depletion is not restricted to the node but spreads along the axon (Figure 2(f) to (g)). The decrease in ATP content is accompanied by an increase in local extracellular potassium concentration reflecting inadequate Na-K-ATPase activity due to local energy depletion (Figure 2(e)). The ion and energy disturbance spreads from the node to adjacent segments being dampened with increasing distance from the damaged node.

### Paranodal demyelination

**Figure 3.**
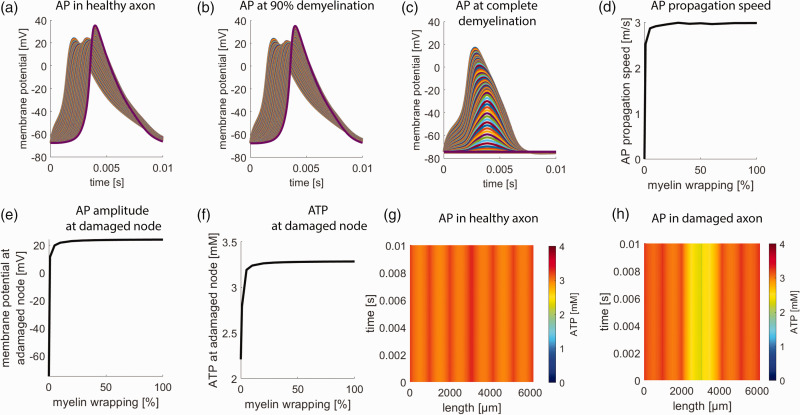
Effects of paranodal demyelination. (a) AP propagation over time in a healthy axon, (b) in an axon with 90% paranodal demyelination, and (c) complete paranodal demyelination around the 3rd NOR. The different curves give the membrane potential of each axon segment. Complete paranodal demyelination leads to a breakdown of AP propagation. (d) AP propagation speed in dependence on the percentage of myelin wrappings around the paranode. At AP breakdown, propagation speed is zero. (e) Maximal axonal membrane potential during AP at the damaged node in dependence on the percentage of myelin wrappings around the paranode. At AP breakdown, there is no depolarization at the damaged node and subsequent axon segments. (f) ATP concentration at the damaged node in dependence on the percentage of myelin wrappings around the paranode. Complete paranodal demyelination leads to decreased ATP availability at the damaged node. (g) Axonal ATP concentration in a healthy axon. ATP concentrations are around 3 mM throughout the whole axon and (h) Axonal ATP concentration in an axon during complete paranodal demyelination at the 3rd NOR. Reduced ATP availability centers around the damaged node and affects adjacent axon segments.

To simulate paranodal demyelination, we successively decreased the degree of myelin wrapping around the affected paranode and juxtaparanode. Figure 3(a) to (c) shows the axonal AP propagation in a healthy axon (Figure 3(a)), an axon with 90% decreased layering (Figure 3(b)), and a completely demyelinated paranode (Figure 3(c)). Progressive paranodal demyelination leads to a reduction in AP propagation velocity from 3 m/s until conduction block. In agreement with Arroyo et al.,^22^ the decrease in paranodal myelin wrapping can be compensated for a long time before functional impairment can be observed. AP propagation block occurred only after complete paranodal demyelination (Figure 3(c)). At this point, local ATP depletion reached values of about 2.2 mM around the demyelinated paranode. Figure 3(d) to (f) shows the dependence of AP propagation velocity, axonal membrane potential, and axonal ATP concentration around the damaged node on the degree of paranodal demyelination. In addition, while ATP concentration is uniform in the healthy axon (Figure 3(g)), paranodal demyelination leads to ATP depletion that is not limited to the demyelinated area but spreads along the axon (Figure 3(g)). Although the demyelinated membrane stretch is much smaller (only 5 µm compared to 50 µm in node widening), the effects are still noticeable. This is because the higher channel density in the juxtaparanodal segment leads to higher leak currents.

### Segmental demyelination

**Figure 4.**
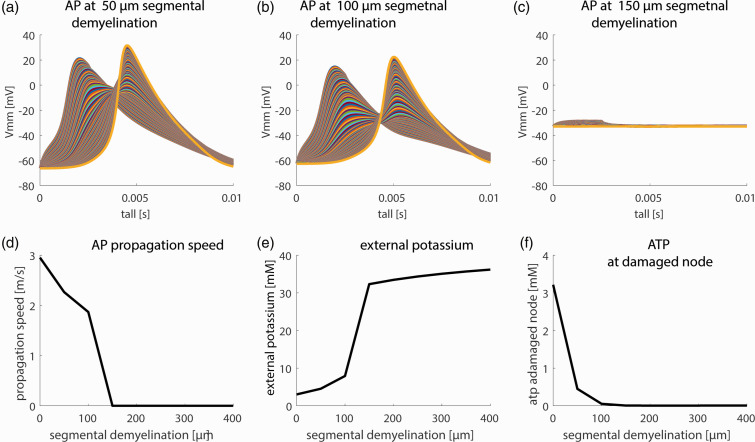
Effects of segmental demyelination. Axonal membrane potential propagation at (a) 50 µm, (b) 100 µm, and (c) 150 µm segmental demyelination at the 3rd NOR. At 150 µm segmental demyelination, the whole axon depolarizes and AP breaks down. (d) AP propagation in dependence on the length of segmental demyelination. (e) Extra-axonal potassium concentration around the damaged node for different lengths of segmental demyelination. In dependence on the length of the demyelinated stretch, ion homeostasis is compromised and (f) ATP concentration at the damaged node in dependence on the length of segmental demyelination. Segmental demyelination leads to severe ATP depletion.

During segmental demyelination, the paranodal and juxtaparanodal structures disappear and the remaining heminode is directly adjacent to the demyelinated membrane stretch, which merges with the myelinated internode segments. We modeled segmental demyelination by a successive increase in the length of the unmyelinated membrane stretch while the paranodal structure was assumed to disappear at the demyelination site. Figure 4 shows the effect of segmental demyelination on axonal AP characteristics and energetic state. Progressive demyelination leads to decreased AP propagation velocity up to a complete breakdown of AP propagation (Figure 4(a) to (d)). This goes along with a decrease in AP amplitude around the demyelinated stretch (Figure 4(e)). As in the case of the paranodal demyelination, we observe severe ATP depletion originating from the demyelinated segments spreading further along the axon as demyelination increases until AP propagation breaks down (Figure 4(g)). This breakdown is associated with a severe shift in resting membrane potential from −70 mV to about −30 mV (Figure 4(a) and (c)) and disruption of ion homeostasis (Figure 4(f)) as the neuron is unable to compensate for the extreme ATP demand associated with membrane demyelination. This leads to a complete breakdown of AP propagation when the demyelinated stretch reaches 150 µm in width (Figure 4(c) and (d)).

### Axonal repair

Segmental demyelination leads to severe impairment of neuronal functionality and metabolic demand that has to be counterbalanced to ensure cellular survival and regain cellular functionality. This repair can occur via metabolic compensation by increased mitochondrial activity in the demyelinated segment (re-energization), and subsequent insertion of ion channels in the demyelinated axon segment.

We simulated axonal re-energization by equipping the demyelinated axon stretches with increasing amounts of glycolytic proteins and mitochondria. The ratio of glycolytic to mitochondrial enzymes was kept constant at the ratio of the AIS – which is not expected to change during demyelination. The total amount of enzymes was successively increased up to 60% of the ATP production capacity of the AIS.

**Figure 5.**
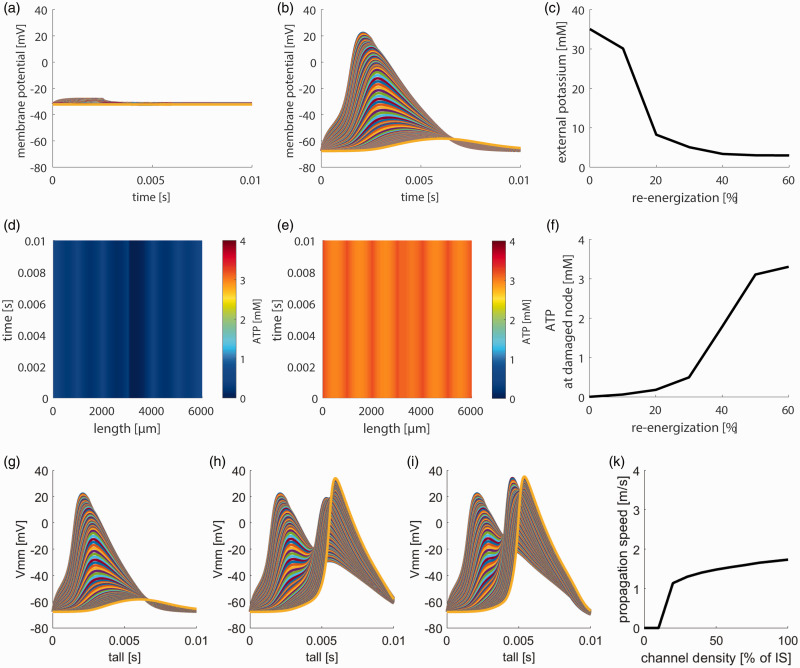
Effect of re-energization and continuous propagation for segmentally demyelination axons. (a) Lack of AP without re-energization. (b) Re-energization corresponding to 50% of the ATP production capacity of the AIS leads to repolarization of the axonal membrane and AP initiation, but there is no AP propagation through the demyelinated membrane stretch. (c) Re-energization decreases extracellular potassium concentration. At re-energization corresponding to 50% of the ATP production capacity of the AIS, ion homeostasis is reestablished. (d) ATP concentration without re-energization. ATP is depleted throughout the axon. (e) With re-energization corresponding to 50% of the AIS, ATP availability is re-established through the whole axon. (f) ATP availability at the damaged node in dependence on re-energization in the percentage of AIS ATP production capacity. A protein density of 50% compared to the AIS reconstitutes ATP availability. (g) AP propagation without additional ion channels. APs can be elicited, but do not propagate through the demyelinated axon stretch. (h) With 50% AIS channel density and (i) 100% AIS channel density. APs propagate through the complete axon, although at different speeds and (k) AP propagation speed in dependence on the degree of channel density. AP propagation speed reaches saturation and cannot be increased by a higher channel density. The resulting AP propagation speed is significantly decreased compared to myelinated axons.

Figure 5(a) to (f) shows the effect of re-energization for a 300 µm segmental demyelinated membrane stretch. Without re-energization, axonal ATP levels are depleted, the axonal membrane is depolarized, and APs cannot be initiated (Figure 5(a) and (d)). By successively increasing the ATP production capacity (Figure 5(e) and (f)), a complete re-energization is reached at 50% of AIS protein densities. This re-energization leads to the repolarization of the axonal membrane (Figure 5(b)) and reconstitution of transmembrane ion distribution (Figure 5(c)). The repolarization of the axonal membrane re-establishes the initiation of AP at the AIS, but as the AP propagates along the axonal membrane, it dissipates at the demyelinated membrane stretch.

After stabilizing ATP levels in a demyelinated axon by increasing the capacity of ATP production, we simulated the regaining of axonal functionality by the insertion of ion channels into the demyelinated stretch. To achieve this, we successively equipped the demyelinated axon segments with ion channels corresponding to 0–100% of the channel densities found in the AIS. Figure 5(h) to (k) shows how the combination of re-energization and ion channel endowments restores neuronal functionality in a 300 µm demyelinated axon. Without additional channels at the demyelinated axonal stretch, APs can be initiated but are not transmitted over the axon (Figure 5(g)). At a channel density corresponding to 50% of the AIS, AP propagation is restored (Figure 5(h)), but AP velocity remains significantly reduced (Figure 5(i) and (k)).

### Pharmacological demyelination

**Figure 6.**
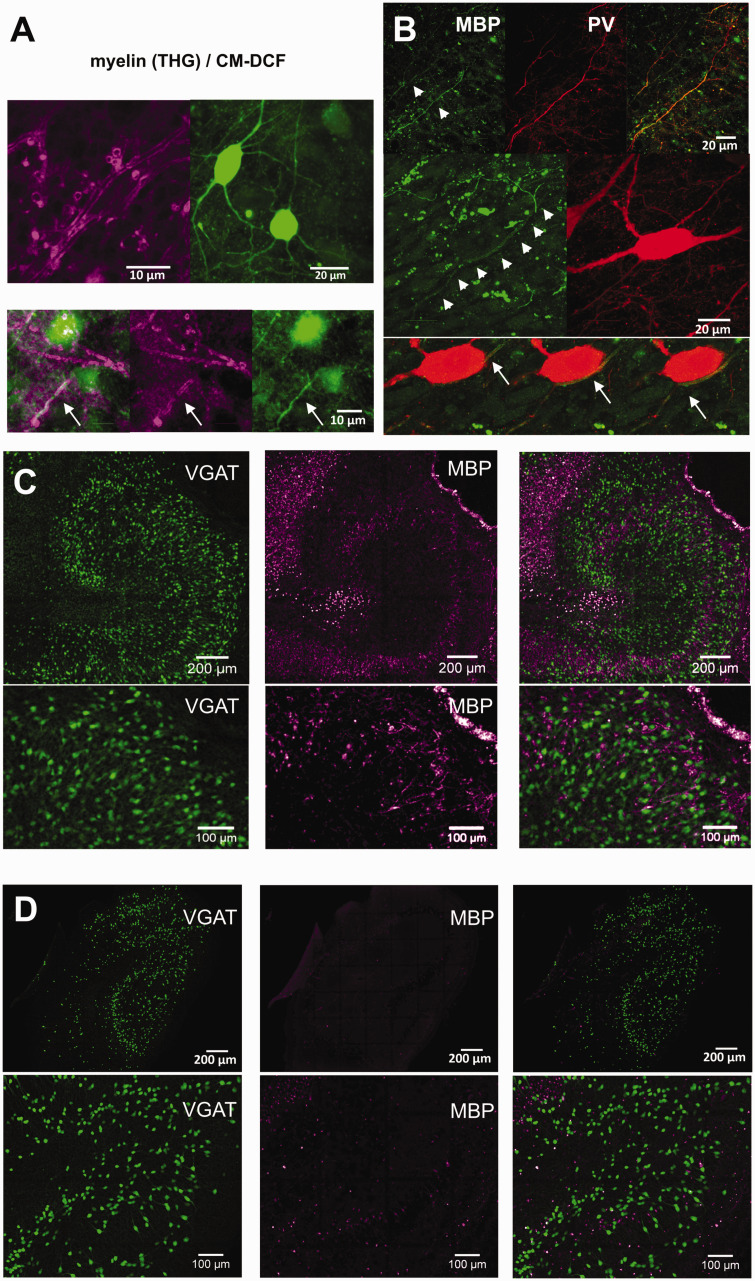
(a) Intrinsic third-harmonic generation (THG) fluorescence microscopy indicated the presence of myelin in slice cultures (upper left). Oxidation of chloromethyl-2,7-dichlorodihydrofluorescein (CM-H_2_DCF) allowed for the identification of metabolically Continued.active fast-spiking interneurons (upper right). While THG was not restricted to DCF+ interneurons, short stretches of DCF+ axons were covered with myelin (lower panels, arrows). (b) Co-labeling of myelin basic protein (MBP, green) and parvalbumin (PV, red). Upper panel: an overview from the border of str. pyramidale/str. lucidum in CA3. Arrowheads indicate thick PV^+^ axons covered with MBP. Middle panel: reconstruction of the soma of a PV^+^ interneuron and an MBP-covered PV^+^ axon passing by. Lower panels: three different focal plains from the same example showing the myelin-covered PV^+^ axon (arrows and arrowheads) crossing beside the soma. (c) Representative examples of yellow fluorescent protein (YFP) positive interneurons (green) from a control, an ethanol (EtOH)-treated, and an EtOH plus lysophosphatidylcholine (LPC)-treated slice culture (left, middle, and right panels, respectively) and (d) Representative examples of MBP and YFP fluorescence in the CA3 region of the slice culture (the MBP fluorescence is represented in false color to enhance visibility). Upper panels: control; lower panels: LPC treated. The LPC treatment did not affect the viability of YFP^+^ interneurons, while the specific MBP fluorescence is absent.

To test the physiological consequences of demyelination we used the simple model system offered by hippocampal slice cultures.^44,48^ Oligodendrocyte precursor cells are present in slice cultures and differentiate into oligodendrocytes creating myelin sheath as revealed by intrinsic THG microscopy *in situ*.^49^ THG arises from compact myelin structures, also enwrapping axons of metabolically active, fast-spiking interneurons, which were identified by the accumulation of the oxidation-sensitive probe, CM-H_2_DCF.^13^ Co-labeling for MBP and PV further substantiated the presence of myelin around PV^+^ axon segments of fast-spiking interneurons in culture (Figure 6(b)). In a previous study, we found that pharmacologically induced gamma oscillations successively increase in power and peak frequency reaching a plateau after the first 10 DIV development.^15^ In addition to the maturation of the fast-spiking interneurons, which occurs in the same time frame, increasing myelination might contribute to the observed changes in neuronal network activity.

Demyelination was achieved by exposure to LPC after 7 DIV for 15–17 hrs.^44^ LPC blocks myelination by interfering with lipid synthesis. As a highly lipophilic drug, LPC has to be solved in organic solvents. We have chosen EtOH, which is non-toxic – as compared to DMSO – in the necessary final concentration. However, EtOH by itself can have an impact on gamma oscillations, therefore, the effects of LPC on gamma oscillations were always compared to gamma oscillations in slice cultures exposed to the same concentration of EtOH without LPC. None of the treatments affected interneuron viability, as revealed by the unaltered density and distribution of YFP-VGAT interneurons (Figure 6(c)). However, MBP immunofluorescence was lost in LPC-treated slice cultures (Figure 6(d)).

### Comparison of oxygen consumption rates following pharmacological demyelination

**Figure 7.**
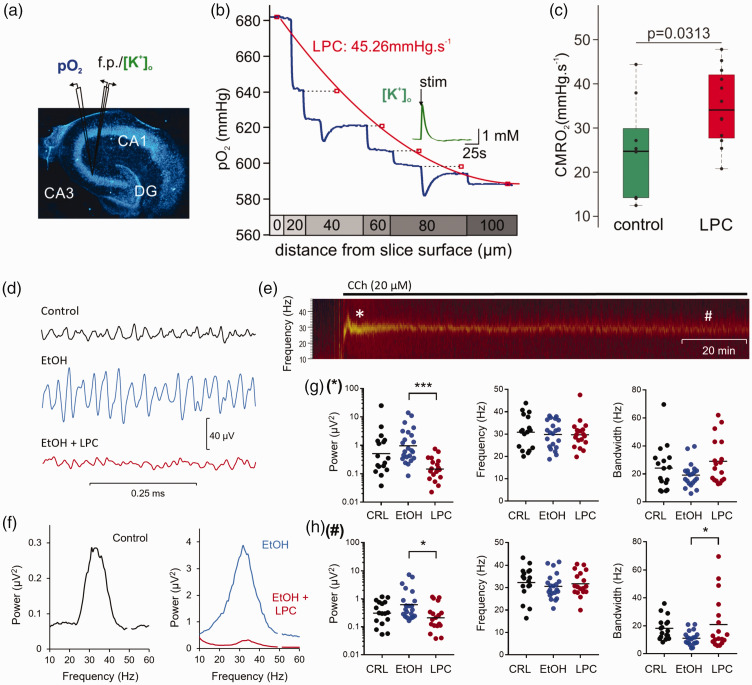
Variations in basal pO_2_ and gamma oscillations under LPC treatment. (a) Left: Picture and scheme of typical electrode placement in hippocampal slice culture. Slice cultures in the interface chamber receive oxygen from the surface and bottom and the partial oxygen pressure (pO_2_) decreases with the distance to the source of oxygen (i.e., distance to the slice bottom and surface) providing typical depth profiles. The oxygen gradient along the slice depends on oxygen supply and solubility, which are both constant under experimental conditions. Hence, changes in pO_2_ depth profiles reflect activity-dependent O_2_ consumption. (b) Using an established reaction-diffusion model, peak pO_2_ levels at each vertical step in the pO_2_ profile were fitted to calculate oxygen consumption rates (OCR). OCRs increased from control (25.0 mmHg · s^−1^) to LPS-treated slices (34.8 mmHg · s^−1^). The insert shows (5 s, 10 Hz) stimulus train-induced changes in pO_2_ and extracellular potassium concentration at a certain depth. (c) The quantitative comparison revealed a significant OCR increase in LPS-treated slices. Black dots depict measurements for individual slices. Box blots depict median and quartiles. The significance level using a two-sided t-test is indicated above. (d) Representative low-pass filtered (150 Hz) original traces of gamma oscillations induced by carbachol (CCh, 20 μM) without pretreatment (control, top), after treatment with EtOH solvent (middle) and LPC (bottom). (e) Sonogram of a gamma oscillation induced by CCh. The oscillations developed within minutes reaching an initial peak after ca. 10 min of application (asterisk). This initial gamma oscillation waned and stabilized at a lower steady-state level after 60 min, which lasted until the end of the experiment (up to 180 min; hashtag). (f) Representative power spectra of gamma oscillations without pretreatment, after treatment with EtOH solvent and LPC. Gamma oscillations after pretreatment with LPC (EtOH + LPC) had a lower power compared to solvent control (EtOH). (g) Peak power (left), peak frequency (middle), and half bandwidth (right) of gamma oscillations during the initial phase of 20 min (marked with an asterisk on the sonogram) without pretreatment (control, CRL), after treatment with EtOH solvent or LPC and (h) Peak power (left), peak frequency (middle), and half bandwidth (right) of gamma oscillations during the steady-state phase after 100 min of induction (marked with a hashtag on the sonogram) without pretreatment (CRL), after treatment with EtOH solvent, or LPC.

Oxygen consumption rates were calculated by applying a mathematical reaction-diffusion model^50^ to experimentally recorded pO_2_ depth profiles (Figure 7(a) and (b)). Under interface recording conditions, pO_2_ was maximal at the slice surface (∼680 mmHg) and minimal (∼580 mmHg) in the slice at a depth of ∼100 µm. After trespassing the core, pO_2_ increased again as oxygen diffused from the bottom via the perfused aCSF. Depth profiles were recorded in slices treated with LPC dissolved in EtOH and the presence of EtOH alone as a control. Figure 7(c) shows that basal oxygen consumption rates increased from 25.0 mmHg · s^−1^ (n = 9, N = 6) under control conditions to 34.83 mmHg · s^−1^ (n = 12, N = 6) for LPC-treated slices indicating a higher ATP consumption upon demyelination.

### Gamma oscillations following pharmacological demyelination

Upon wash-in of CCh (20 μM), hippocampal slice cultures developed gamma oscillations within minutes (Figure 7(e)) reaching a peak power of 0.51 µV^2^ (95% CI: 0.22, 1.2; n = 17; N = 11). This initial gamma oscillation waned and stabilized after 60 min at lower steady-state power of 0.31 µV^2^ (95% CI: 0.18, 0.51), which lasted until the end of the measurement (170 min). EtOH-treated slice cultures presented a somewhat higher increase in gamma power compared to untreated control slices in both the initial (0.97 µV^2^; 95% CI: 0.54, 1.72; n = 23; N = 11; p = 0.503 compared to control) and steady-state phase (0.62 µV^2^, 95% CI: 0.37, 1.04; p = 0.565; Figure 7). By contrast, LPC application significantly decreased the gamma power during both phases compared to EtOH (initial phase: 0.15 µV^2^; 95% CI: 0.09, 0.23; n = 18; N = 10; p < 0.0001; steady-state phase: 0.21 µV^2^; 95% CI: 0.12, 0.36; p = 0.028). Remarkably, the peak frequency of the gamma oscillations remained unaltered (p = 0.997 and 0.837 during the initial and steady-state phase, respectively), but the half-bandwidth of the oscillations significantly increased in LPC-treated slice cultures in the steady-state phase (20.9 ± 18.9 Hz; 95% CI: 11.5, 30.3; p = 0.040) compared to EtOH (11.1 ± 4.9 Hz; 95% CI: 8.8, 13.4). The reciprocal relationship between bandwidth and peak power (i.e. decreasing power with increasing bandwidth) is a sign of disturbed synchronization of neuronal ensembles.^51,52^

## Discussion

Current models of axonal demyelination (see Coggan et al.^25^ for a comprehensive review) make use of electric circuit analogs of differing complexity and are very successful in describing electrophysiological phenomena like decreased AP speed or conduction block. However, functional impairment during demyelination is not limited to aberrant AP propagation but includes energy depletion and the disruption of ion homeostasis.^9,25^ These phenomena could not be addressed as previous models lack the explicit description of ion movement and the associated energy expenditure needed for ion transport.

To study the metabolic effects of demyelination-related axonal defects, we developed a comprehensive model, which combines ion movement through voltage-gated and basal leak channels with a detailed physiological model of neuronal energy metabolism. Instead of using cable theory, in the present model, the changes in the axonal membrane potential in an axon segment result from ion movement across the membrane of that segment and electrical coupling between adjacent axonal segments. These innovations enable the explicit coupling of electrophysiological processes to neuronal ATP consumption rate through the activity of the Na-K-ATPase and allow for quantitative assessment of the metabolic changes during different stages of demyelination disease. A key finding of the simulations is the severe compromise of energy efficiency of AP propagation and the subsequent decrease in cellular ATP levels occurring in all forms of demyelination.

During nodal widening and paranodal demyelination, intra-axonal ATP levels can decrease to ∼20% and ∼60% of the control values around the damaged membrane stretches, but ATP content at the AIS and the AT remains intact. Despite the ‘local’ disruption of energy provision, axonal resting membrane potential remains stable and both, the generation of AP at the AIS and even the propagation across the damaged node, are still possible. The decrease in AP propagation velocity is a consequence of the altered conduction properties at the damaged segments but is not directly related to energy depletion. Therefore, solely electrophysiological models can be used to describe the effects of nodal widening and paranodal demyelination.^26^ Regarding paranodal demyelination, our model suggested that the loss of up to 90% of the initial myelin wraps can be still compensated before a breakdown of the conductance occurs. This is in good agreement with previous models based on electrical circuit analogs, showing that changes in the conduction velocity take only place when the myelin sheet is already severely decreased. Action potentials were blocked when the myelin thickness is reduced to less than 2% of its original value.^53^ With a loss of myelin wrappings as severe as 80% of the initial thickness, this model suggested only a 13% decrease in conduction velocity, which is in good agreement with our results despite the differences in the model.

A direct consequence of energy depletion is the local increase in extracellular potassium. Around the damaged nodal segment, the leakage of ions is increased while the activity of the Na-K-ATPase is compromised due to energy depletion. This leads to a shift in transmembrane ion distribution that is only partially dampened by diffusion into adjacent segments.

Segmental demyelination represents the advanced state of demyelination disease with severely compromised axonal functionality. If the demyelinated membrane stretch is sufficiently small (∼50 µm), segmental demyelination resembles nodal widening with decreased AP propagation velocity, partial energetic breakdown, and local disruption of ion homeostasis. However, if the demyelinated area increases to >150 µm, a complete AP block occurs along with global energy depletion and a severe increase in extracellular potassium.

While our model considers only a single axon disregarding the effect of neighboring cells, it is plausible to assume that changes in the ion distribution resulting from severe demyelination will add up and might eventually override the spatial buffer capacity of astrocytes and oligodendrocytes. Extracellular potassium accumulation is associated with multiple forms of hyper-excitability.^33,54,55^ In line with this finding, Felts et al.^56^ report on the ectopic firing in demyelinated axons resembling the activity following potassium loading of extracellular space in myelinated fibers and suggest that an elevated periaxonal potassium concentration may occur within compartments in demyelinating lesions and that it can result in the generation of ectopic impulse. Thus, our model can explain phenomena such as pathological ephaptic coupling or the generation of ectopic APs occurring in multiple sclerosis, Guillain Barré syndrome, trigeminus neuralgia, facial spasms, or ocular neuromyotonia.^56,57^

Finally, the calculations showed that conduction recovery in demyelinated axons can be achieved by the insertion of voltage-gated ion channels in the demyelinated membrane. This is in line with several studies that demonstrated the spread of nodal membrane characteristics through demyelinated axon regions.^30,31^

We used two different strategies to validate our modeling data. First, we measured oxygen consumption after demyelination and found that consumption rates significantly increased suggesting that demyelination and resulting compensatory axon re-energization and remodeling from saltatory to continuous conductivity greatly increase energy demand in the affected regions. These changes would represent an effect of demyelination regardless of the cell type i.e. interneurons or pyramidal cells.

Second, we used gamma oscillations as a sensible biomarker to investigate the effect of demyelination on PV^+^ interneurons. Assuming that myelination is critical for precise timing along the widespread axon arbor and minimizing the energy demand of high-frequency firing, demyelination will have a profound effect on the perisomatic inhibition and thus on the power of gamma oscillations.^8,11,58^ Energy deficiency of PV^+^ basket cells was shown to lead to disturbances in social behavior and the underlying network activity.^59^ In a recent study, optogenetically induced gamma oscillations in cuprizone-treated demyelinated mice were impaired along with a reduction in phasic GABA release and increased the failure rate of coupled PV^+^ interneuron–pyramidal cell pairs.^60^ Interestingly, while changes in conduction velocity at PV^+^ axons were not observed following cuprizone treatment, a reduced conduction velocity was found in a transgenic mouse model with aberrant myelination patterns i.e. increased internode length and node widening.^61^ Our experiments confirm the results of Dubey et al.^60^ and show that demyelination disturbs energy homeostasis under basal conditions and disrupts the synchronization of neuronal networks in the gamma frequency range. As the power of local field potential oscillations is determined by the precision of synchronous inhibition of the PV^+^ basket cell network entraining the activity of pyramidal cells,^18^ the observed decrease in the power and widening of the bandwidth upon demyelination indicates a disturbance in the synchronization following demyelination (Figure 7(d) to (h)). Changes in myelination were shown to alter network activities different from gamma oscillations. Genetic deficit of the myelin disturbed both spontaneous and movement-related neural activity in the motor cortex by decreasing conduction velocity and synchrony of thalamocortical long-range inputs.^62^ Inhibition of the *de novo* myelination decoupled sharp wave ripples and cortical spindles indicating that oligodendrogenesis can alter synchronous hippocampal-cortical networks.^63^ We conclude that computational modeling of demyelination has to consider the metabolic aspects in addition to the electrophysiological effects of myelin loss as these can profoundly impact system behavior. The model presented here additionally allows for testing hypotheses regarding disturbances in axonal mitochondria^64^ as well as the supportive role of myelin for axonal development and function.^65,66^

Limitations of the model: This work focused on the demyelination-induced changes in metabolic demand and accompanying metabolic limitations. However, the real situation is more complex than that. Myelin is not only important for isolation and therefore for significant reduction of metabolic demand of axons but provides axons with lactate as fuel for oxidative phosphorylation and thereby actively satisfies metabolic demand. This role of myelin is disregarded in the model, as substrate supply is assumed to be not rate-limiting. Therefore, the metabolic consequences of axonal demyelination might even be more aggravating in real-life situations. For simplicity, we also assume that there is no space for ion exchange between the axon and the innermost myelin sheath, thereby neglecting possible local changes in ion concentrations occurring in the space between the axon and the myelin that might additionally impact AP propagation. A further simplifying assumption relates to the distribution of ATP production and utilization along the axon. We assume that ATP production and consumption are mainly located at the NOR, but there is evidence that ATP production and consumption also occur at the internode, paranode, and juxtaparanode. Likewise, ATP consumption is not only limited to electrophysiological processes as assumed in our model but other ATP consumers (e.g. basal protein turnover and structural maintenance) account for about 30% of overall ATP consumption. To check that these assumptions do not compromise the overall model characteristics, we repeated all simulations with an additional unspecific, high-affinity ATP consumer adding 30% to the basal ATP consumption rate. This amounts to an overall decrease in ATP levels by ∼15% but maintains all general features of the model (see Supplementary Figures S2–S5).

## Supplemental Material

sj-pdf-1-jcb-10.1177_0271678X231170746 - Supplemental material for Metabolic implications of axonal demyelination and its consequences for synchronized network activity: An *in silico* and *in vitro* study Click here for additional data file. Supplemental material, sj-pdf-1-jcb-10.1177_0271678X231170746 for Metabolic implications of axonal demyelination and its consequences for synchronized network activity: An *in silico* and *in vitro* study by Zoltan Gerevich, Richard Kovács, Agustin Liotta, Luisa A Hasam-Henderson, Ludwig Weh, Iwona Wallach and Nikolaus Berndt in Journal of Cerebral Blood Flow & Metabolism

sj-pdf-2-jcb-10.1177_0271678X231170746 - Supplemental material for Metabolic implications of axonal demyelination and its consequences for synchronized network activity: An *in silico* and *in vitro* study Click here for additional data file. Supplemental material, sj-pdf-2-jcb-10.1177_0271678X231170746 for Metabolic implications of axonal demyelination and its consequences for synchronized network activity: An *in silico* and *in vitro* study by Zoltan Gerevich, Richard Kovács, Agustin Liotta, Luisa A Hasam-Henderson, Ludwig Weh, Iwona Wallach and Nikolaus Berndt in Journal of Cerebral Blood Flow & Metabolism
