# Supplementary material for: Metabolic implications of axonal demyelination and its consequences for synchronized network activity: An in silico and in vitro study
Source: J Cereb Blood Flow Metab. 2023 Apr 26;43(9):1571–87. doi: 10.1177/0271678X231170746 (PMC10414014; doi:10.1177/0271678X231170746)
Supplement: sj-pdf-1-jcb-10.1177_0271678X231170746 - Supplemental material for Metabolic implications of axonal demyelination and its consequences for synchronized network activity: An in silico and in vitro study [file sj-pdf-1-jcb-10.1177_0271678X231170746.pdf]

Supplement

Table of Contents

Electrophysiological Model 3
Metabolic Model 7
Glycolysis 7
Glucose transporter 7
Hexokinase 7
Glucose-6-phosphate isomerase 7
Phosphofructokinase I 8
Fructose-1,6-bisphosphatase 9
Phosphofructokinase II 9
Fructose-2,6-biphosphatase 9
Aldolase 10
Triose phosphate isomerase 10
Glycerinaldehyde-3-phosphate dehydrogenase 10
Phosphoglycerate kinase 11
Phosphoglycerate mutase 11
Enolase 12
Pyruvate kinase 12
Lactate dehydrogenase 13
Monocarboxylate transporter (MCT) 13
Adenylatkinase 13
Creatin kinase 14
Malate dehydrogenase (cytoplasmic) 14
Stoichiometric matrix 14
Citric Acid Cycle and Redox Shuttle Systems 16
Pyruvate transporter (mitochondrial membrane) 16
Pyruvate dehydrogenase complex 16
Citrate synthetase 17
Aconitase 17
Isocitrate dehydrogenase (NAD-dependent) 18
Alpha-ketogluterate dehydrogenase 19

Succinyl-CoA synthetase 20
Succinate dehydrogenase 21
Fumarase 21
Malate dehydrogenase (mitochondrial) 22
Nucleoside diphosphokinase (mitochondrial) 22
Aspartate amino transferase 23
Aspartate amino transferase (mitochondial) 23
Aspartate-glutamate transporter 23
Malate-α-ketoglutarate transporter 24
Glycerol-3-phosphate dehydrogenase (cytosolic) 24
Glycerol-3-phosphate dehydrogenase (mitochondrial) 24
Stoichiometric matrix 25
Respiratory Chain and Mitochondrial Electrophysiology 27
Mitochondrial ATP exchanger [77] 27
ATP synthetase 27
Complex I 28
Complex III 28
Complex IV 29
Oxygen diffusion 29
Pump currents 29
Proton leak 30
Sodium leak 30
Potassium leak 30
Chloride leak 30
Calcium–proton exchanger 30
Calcium–sodium exchanger 31
Calcium currents 31
ATP-dependent cytosolic calcium increase 32
Stoichiometric matrix 32
External conditions 33
References 34

Electrophysiological Model

Changes in ion concentrations for chloride, sodium, and potassium are given by the sum of local transmembrane ion currents I_X and diffusive currents between adjacent compartments I_X^{diff} ($X = \{Na, K, Cl\}$):

$$\frac{d[X_{cell}]}{dt} = -\frac{I_X}{z_X F \Omega_{cell}} + I_X^{diff_{in}}$$

$$\frac{d[X_{out}]}{dt} = \frac{I_X}{z_X F \Omega_{out}} + I_X^{diff_{out}}$$

Transmembrane currents are the sum of active currents I_X^a and passive currents I_X^p

$$I_X = I_X^a + I_X^p$$

Passive ion currents are given by the Goldman-Hodgkin-Katz equation:

$$I_X^p = -A g_X U F z_X^2 \frac{[X_{cell}]\exp(z_X U) - [X_{out}]}{1 - (\exp(z_X U))},$$

with U given by $U = v_{mm} \cdot \frac{F}{1000 \cdot RT}$, $F = 96490 \frac{C}{mol}$, $R = 8.314 \frac{J}{K \cdot mol}$, $T = 310\,K$.

The permeability is the sum of basal, gated, and excitatory permeability:

$$g_X = P_X^0 + P_X^g + P_X^{exc}$$

Gated permeability exists only in the axon initial segment and is modeled according to [1]:

$$P_K^g = m^4 P_K^{g0}$$

$$P_{Na}^g = n^3 h P_{Na}^{g0}$$

$$\frac{dg}{dt} = C_g\left(\alpha_g(1-g) + \beta_g\right)$$

Diffusive currents are given by:

$$I_X^{diff_{in}} = -\frac{D_{in}}{Vol_{in}^i}\left(\frac{A_{i-1}}{l_{i-1}}\left(X^i - X^{i-1}\right) + \frac{A_i}{l_i}\left(X^i - X^{i+1}\right)\right)$$

$$I_X^{diff_{out}} = -\frac{D_{ext}}{Vol_{ext}^i}\left(\frac{A_{i-1}}{l_{i-1}}\left(X_{ext}^i - X_{ext}^{i-1}\right) + \frac{A_i}{l_i}\left(X_{ext}^i - X_{ext}^{i+1}\right)\right) \qquad for\ 1 < i < n,$$

with i labeling the different segments of the axon, i+1 and i-1 being the right and left neighboring segments of segment i.

Changes in membrane potential due to ion currents in any segment are given by the sum of changes due to transmembrane currents and coupling between adjacent compartments:

$$\frac{dV}{dt} = \frac{1}{C}\sum_{X} I_X \quad + v_V^{diff}$$

Total capacitance C is given by:

$$C = c_m A = \frac{Q}{V}$$

Coupling of membrane potential between adjacent segments is given by:

$$v_V^{diff} = + \frac{\Delta V}{\frac{R_{ax} \cdot (l_{i-1} + l_i)/2}{\pi r^2}}$$

With $\Delta V = (V_{i+1} - V_i) + (V_i - V_{i-1}) \qquad for\, 1 < i < n.$

Ion permeabilities are the sum of basal permeabilities P_X^0 and Hodgkin-Huxley like permeabilities of voltage-gated channels P_X^g.

$$g_{Na} = P_{Na}^0 + m^3 h P_{Na}^g$$

$$P_{Na}^0 = \begin{bmatrix} node: & 1.2 \\ paranode: & 1.2 \cdot f_m \\ juxtaparanode: & 1.2 \cdot f_m \\ internode: & 1.2 \cdot f_m \\ AIS: & 1.2 \\ AT: & 1.2 \end{bmatrix} \cdot 10^{-9} \frac{m}{s} \qquad [2]$$

$$P_{Na}^g = \begin{bmatrix} node: & 1500 \\ paranode: & 0 \\ juxtaparanode: & 0 \\ internode: & 0 \\ AIS: & 1500 \\ AT: & 1500 \end{bmatrix} \cdot 10^{-9} \frac{m}{s} \qquad [3]$$

$$g_K = P_K^0 + \ n^4 P_K^g$$

$$P_K^0 = \begin{bmatrix} node: & 17 \\ paranode: & 17 \cdot f_m \\ juxtaparanode: & 17 \cdot f_m \\ internode: & 17 \cdot f_m \\ AIS: & 17 \\ AT: & 17 \end{bmatrix} \cdot 10^{-9} \qquad [2]$$

$$P_K^g = \begin{bmatrix} node: & 225 \\ paranode: & 0 \\ juxtaparanode: & 0 \\ internode: & 0 \\ AIS: & 225 \\ AT: & 225 \end{bmatrix} \cdot 10^{-9}\,\frac{m}{s} \qquad [3]$$

$$g_{Cl} = P_{Cl}^0$$

$$P_{Cl}^0 = \begin{bmatrix} node: & 20 \\ paranode: & 20 \cdot f_m \\ juxtaparanode: & 20 \cdot f_m \\ internode: & 20 \cdot f_m \\ AIS: & 20 \\ AT: & 20 \end{bmatrix} \cdot 10^{-9}\,\frac{m}{s} \qquad [2]$$

$$f_m = \frac{1}{n_{myelin} + 1}$$

Hodgkin-Huxley transition rates are taken from [4], given by :

$$C_m = 1000$$

$$C_n = 1000$$

$$C_h = 1000$$

$$\alpha_m = \frac{0.1 - 0.01(V + 60)}{\exp\big(1.0 - 0.1(V + 60)\big) - 1.0}$$

$$\alpha_n = \frac{2.5 - 0.1(V + 65)}{\exp\big(2.5 - 0.1(V + 65)\big) - 1}$$

$$\alpha_h = 0.07\exp\left(\frac{-(V + 65)}{20}\right)$$

$$\beta_m = 0.125\exp\left(\frac{-(V + 65)}{80}\right)$$

$$\beta_n = 4.8\exp\left(\frac{-(V + 65)}{18}\right)$$

$$\beta_h = \left(\exp\left(\frac{30 - (V + 75)}{10}\right) + 1\right)^{-1}$$

Active ion currents are given by the NaK-ATPase:

$$I_X^{ATPase} = a_X \cdot A \cdot \frac{atp}{atp + K_m^{atp}} \cdot \frac{na^{n_{na}}}{na^{n_{na}} + (K_m^{na})^{n_{na}}} \cdot \frac{k_{ext}^{n_k}}{k_{ext}^{n_k} + \big(K_m^k\big)^{n_k}}$$

$K_m^{atp} = 0.3$ mM [5]

$n_{na} = 4$ mM [5]

$K_m^{na} = 10$ mM [5]

$n_k = 2$ [5]

$K_m^k = 5$ mM [5]

$with\ X = Na, K \quad and \quad a_{Na} = -3,\ \ a_K = 2$

Changes in ATP, ADP, and phosphate rates are given by:

$$v_{atp} = -\frac{I_{NaKP}}{F \cdot Vol_{in}} \cdot 10^6$$

$$v_{adp} = \frac{I_{NaKP}}{F \cdot Vol_{in}} \cdot 10^6$$

$$v_p = \frac{I_{NaKP}}{F \cdot Vol_{in}} \cdot 10^6$$

Capacitance, axonal resistance, and axonal geometry are given by:

$$C_m = \begin{bmatrix} node: & 1 \\ paranode: & 1 \cdot f_m^c \\ juxtaparanode: & 1 \cdot f_m^c \\ internode: & 1 \cdot f_m^c \\ AIS: & 1 \\ AT: & 1 \end{bmatrix} \frac{\mu F}{cm^2}$$ [1]

$$f_m^c = \frac{1}{\log\left(\frac{r_{ax}+r_{myelin}}{r_{ax}}\right)}$$ [6]

$R_{ax} = 50\ \Omega cm$ [6, 7]

$$l = \begin{bmatrix} node: & 5 \\ paranode: & 3 \\ juxtaparanode: & 50 \\ internode: & 900 \\ AIS: & 20 \\ AT: & 40 \end{bmatrix} \mu m$$ [8]

$r = 1\ \mu m$ [6]

Extracellular space is assumed to be 30% of the cellular space ([4] and references within).

Metabolic Model

Glycolysis

Rate equations for enzymes and transporters are given by:

Glucose transporter

($glc_{ext} \leftrightarrow glc$)

$$v_{glcT} = v_{max}^{glcT} \cdot \frac{glc_{ext} - glc}{1 + \frac{glc_{ext}}{K_m^{glc_{ext}}} + \frac{glc}{K_m^{glc}}}$$

$v_{max}^{glcT} = 0.08\ \mu mol/g/s$

$K_m^{glc_{ext}} = 2.87$ mM [9]

$K_m^{glc} = 2.87$ mM [9]

Hexokinase

($Glc + ATP \rightarrow Glc6P + ADP$)

$$v_{hk} = v_{max}^{hk} \cdot \frac{glc}{glc + K_m^{glc}} \cdot \frac{atp}{atp + K_m^{atp} \cdot \left(1 + \frac{glc6p}{K_{i-atp}^{glc6p}}\right)} \cdot \left(1 - \frac{glc6p}{K_i^{glc6p}}\right)$$

$v_{max}^{hk} = 0.18\ \mu mol/g/s$

$K_m^{atp} = 0.37$ mM [10]

$K_{i-atp}^{glc6p} = 0.074$ mM [11]

$K_i^{glc6p} = 0.1$ mM [10]

$K_m^{glc} = 0.043$ mM [10]

Glucose-6-phosphate isomerase

($glc6p \leftrightarrow fru6p$)

$$v_{gpi} = v_{max}^{gpi} \cdot \frac{glc6p - \frac{fru6p}{K_{eq}^{gpi}}}{1 + \frac{glc6p}{K_m^{glc6p}} + \frac{fru6p}{K_m^{fru6p}}}$$

$v_{max}^{gpi} = 1.66\ \mu mol/g/s$

$K_{eq}^{gpi} = 0.3$ mM [12]

$K_m^{glc6p} = 0.593$ mM [13]

$K_m^{fru6P} = 0.095$ mM [13]

Phosphofructokinase I

(fru6p + atp → fru16p + adp)

$$v_{pfk1} = v_{max}^{pfk1} \cdot \frac{fru6p}{fru6p + K_m^{fru6p}} \cdot \frac{atp}{atp + K_m^{atp}} \cdot \left(1 - \frac{atp^n}{atp^n + \left(K_i^{atp}\right)^n}\right) \cdot \frac{fru26p}{fru26p + K_{a2}^{fru26p}}$$

$v_{max}^{pfk1} = 3.38\ \mu mol/g/s$

$$K_m^{fru6p} = K_0^{fru6p} \cdot \left(1 - f_a^{fru26p} \cdot \left(\frac{fru26p^{n1}}{fru26p^{n1} + \left(K_{a1}^{fru26p}\right)^{n1}}\right)\right)$$

$K_0^{fru6p} = 0.111$ mM [14]

$f_a^{fru26p} = 0.55$ mM [14]

$n1 = 5.5$ [14]

$K_{a1}^{fru26p} = 0.0042$ mM [14]

$K_m^{atp} = 0.04$ mM [15]

$K_i^{atp} = 1.2$ mM [15]

$n = 1.8$ [15]

$K_a^{Fru26P} = 0.005$ mM [15]

Fructose-1,6-bisphosphatase

(Fru16P → Fru6P + Pi)

$$v_{fbp1} = v_{max}^{fbp1} \cdot \frac{fru16p}{fru16p + K_m^{fru6p}}$$

$v_{max}^{fbp1} = 0.32\ \mu mol/g/s$

$K_m^{fru6P} = 0.132$ mM [16]

Phosphofructokinase II

(Fru6P + ATP → Fru26P + ADP)

$$v_{pfk2} = v_{max}^{pfk2} \cdot \frac{fru6p}{fru6p + K_m^{fru6p}} \cdot \frac{atp}{atp + K_m^{atp}} \cdot \frac{adp}{adp + K_a^{adp}} \cdot \frac{amp}{amp + K_a^{amp}}$$

$v_{max}^{pfk2} = 1.82 \cdot 10^{-4}\ \mu mol/g/s$

$K_m^{fru6p} = 0.027$ mM [17]

$K_m^{atp} = 0.055$ mM [17]

$K_a^{adp} = 0.056$ mM [18]

$K_a^{amp} = 0.073$ mM [19]

Fructose-2,6-biphosphatase

(fru26p → fru6p + p)

$$v_{fru26bp} = v_{max}^{fru26bp} \cdot \frac{fru26p}{fru26P + K_m^{fru26p} \cdot \left(1 + \frac{fru6p}{K_i^{fru6p}}\right)}$$

$v_{max}^{fru26bp} = 2.5 \cdot 10^{-3}\ \mu mol/g/s$

$K_m^{fru26p} = 0.07$ mM [17]

$K_i^{fru6p} = 0.02$ mM [17]

Aldolase

(fru16p ↔ grap + dhap)

$$v_{ald} = v_{max}^{ald} \cdot \frac{fru16p - \frac{1}{K_{eq}^{ald}} grap \cdot dhap}{1 + \frac{fru16p}{K_m^{fru16p}} + \left(1 + \frac{grap}{K_m^{grap}}\right)\left(1 + \frac{dhap}{K_m^{dhap}}\right) - 1}$$

$v_{max}^{ald} = 25.48\ \mu mol/g/s$

$K_{eq}^{ald} = 0.0976$ mM [20]

$K_m^{fru16p} = 0.003$ mM [21]

$K_m^{grap} = 0.08$ mM [21]

$K_m^{dhap} = 0.03$ mM [21]

Triose phosphate isomerase

(dhap ↔ grap)

$$v_{tpi} = v_{max}^{tpi} \cdot \frac{dhap - \frac{grap}{K_{eq}^{tpi}}}{\left(1 + \frac{dhap}{K_m^{dhap}} + \frac{grap}{K_m^{grap}}\right)}$$

$v_{max}^{tpi} = 6.37 \cdot 10^4\ \mu mol/g/s$

$K_{eq}^{tpi} = 0.0545$ mM [20]

$K_m^{dhap} = 0.84$ mM [22]

$K_m^{grap} = 1.65$ mM [22]

Glycerinaldehyde-3-phosphate dehydrogenase

(grap + p + nad → bpg13 + nadh)

$$v_{gapdh} = v_{max}^{gapdh} \cdot \frac{\left(nad \cdot grap \cdot p - bpg13 \cdot \frac{nadh}{K_{eq}^{gapdh}}\right)}{\left(1 + \frac{nad}{K_m^{nad}}\right)\left(1 + \frac{grap}{K_m^{grap}}\right)\left(1 + \frac{p}{K_m^{p}}\right) + \left(1 + \frac{bpg13}{K_m^{bpg13}}\right)\left(1 + \frac{nadh}{K_m^{nadh}}\right) - 1}$$

$v_{max}^{gapdh} = 910\ \mu mol/g/s$

$K_{eq}^{gapdh} = 0.0868$ mM [23]

$K_m^{nad} = 0.01$ mM [24]

$K_m^{grap} = 0.101$ mM [24]

$K_m^{p} = 3.9$ mM [25]

$K_m^{bpg13} = 0.0035$ mM [26]

$K_m^{nadh} = 0.008$ mM [25]

Phosphoglycerate kinase

(bpg13 + adp ↔ pg3 + atp)

$$v_{pgk} = v_{max}^{pgk} \cdot \frac{bpg13 \cdot adp - \frac{1}{K_{eq}^{PGK}} \cdot pg3 \cdot atp}{\left(1 + \frac{bpg13}{K_m^{bpg13}}\right)\left(1 + \frac{adp}{K_M^{ADP}}\right) + \left(1 + \frac{pg3}{K_M^{Pg3}}\right)\left(1 + \frac{atp}{K_M^{ATP}}\right) - 1}$$

$v_{max}^{pgk} = 189\ \mu mol/g/s$

$K_{eq}^{pgk} = 1310$ mM [27]

$K_m^{bpg13} = 0.063$ mM [28]

$K_m^{adp} = 0.42$ mM [28]

$K_m^{pg3} = 0.67$ mM [28]

$K_m^{atp} = 0.25$ mM [28]

Phosphoglycerate mutase

(pg3 ↔ pg2)

$$v_{pgm} = v_{max}^{pgm} \cdot \frac{pg3 - \frac{pg2}{K_{eq}^{pgm}}}{\left(1 + \frac{pg3}{K_m^{pg3}}\right) + \left(1 + \frac{pg2}{K_m^{pg2}}\right) - 1}$$

$v_{max}^{pgm} = 182\ \mu mol/g/s$

$K_{eq}^{pgm} = 0.1814$ mM [29]

$K_m^{pg3} = 0.22$ mM [30]

$K_m^{pg2} = 0.28$ mM [31]

Enolase

(pg2 ↔ pep)

$$v_{eno} = v_{max}^{eno} \cdot \frac{pg2 - \frac{pep}{K_{eq}^{eno}}}{1 + \frac{pg2}{K_m^{pg2}} + 1 + \frac{pep}{K_m^{pep}} - 1}$$

$v_{max}^{eno} = 2730\ \mu mol/g/s$

$K_{eq}^{eno} = 0.5$ mM [32]

$K_m^{pg2} = 0.05$ mM [33]

$K_m^{pep} = 0.15$ mM [33]

Pyruvate kinase

(pep + adp ↔ pyr + atp)

$$v_{pk} = v_{max}^{pk} \cdot \frac{pep}{pep + K_m^{pep}} \cdot \frac{adp}{adp + K_m^{adp} \cdot \left(1 + \frac{atp}{K_i^{atp}}\right)}$$

$v_{max}^{pk} = 0.3\ \mu mol/g/s$

$K_m^{pep} = 0.074$ mM [34]

$K_m^{adp} = 0.42$ mM [35]

$K_i^{atp} = 4.4$ mM [35]

Lactate dehydrogenase

(pyr + nadh ↔ lac + nad)

$$v_{ldh} = v_{max}^{ldh} \cdot \frac{pyr \cdot nadh - \frac{1}{K_{eq}^{ldh}} \cdot lac \cdot nad}{\left(1 + \frac{pyr}{K_m^{pyr}}\right)\left(1 + \frac{nadh}{K_m^{nadh}}\right) + \left(1 + \frac{lac}{K_m^{lac}}\right)\left(1 + \frac{nad}{K_M^{nad}}\right) - 1}$$

$v_{max}^{ldh} = 1.3 \cdot 10^4\ \mu mol/g/s$

$K_{eq}^{ldh} = 8400$ mM [36]

$K_m^{pyr} = 0.36$ mM [37]

$K_m^{nadh} = 0.043$ mM [38]

$K_m^{lac} = 4.2$ mM [37]

$K_m^{nad} = 0.088$ mM [39]

Monocarboxylate transporter (MCT)

(lac ↔ lac_{ext})

$$v_{lacT} = v_{mx}^{lacT}\left(\frac{lac - lac_{ext}}{\left(1 + \frac{lac}{K_m^{lac}}\right) + \left(1 + \frac{lac_{ext}}{K_m^{lac_{ext}}}\right) - 1}\right)$$

$v_{mx}^{lacT} = 0.13\ \mu mol/g/s$

$K_m^{lac} = 1.1$ mM [40]

$K_m^{lac_{ext}} = 1.1$ mM [40]

Adenylatkinase

(2 adp ↔ atp + amp)

$$v_{adk} = v_{max}^{adk} \cdot (adp^2 - \frac{amp \cdot atp}{K_{eq}^{adk}})$$

$v_{max}^{adk} = 1.3\ \mu mol/g/s$

$K_{eq}^{adk} = 1$ mM [41]

Creatin kinase

(cr + atp ↔ crp + adp)

$$v_{ck} = -v_{max}^{ck} \cdot \left(atp \cdot cr - \frac{adp \cdot crp}{K_{eq}^{ck}}\right)$$

$v_{max}^{ck} = 0.13\ \mu mol/g/s$

$K_{eq}^{ck} = 0.14$ mM [42]

Malate dehydrogenase (cytoplasmic)

(mal + nad ↔ oaa + nadh)

$$v_{mdh} = v_{max}^{mdh} \cdot \frac{mal \cdot nad - \frac{1}{K_{eq}^{mdh}} \cdot oaa \cdot nadh}{\left(1 + \frac{mal}{K_m^{mal}}\right)\left(1 + \frac{nad}{K_m^{nAD}}\right) + \left(1 + \frac{oaa}{K_m^{oaa}}\right)\left(1 + \frac{nadh}{K_m^{nadh}}\right) - 1}$$

$v_{max}^{mdh} = 1300\ \mu mol/g/s$

$K_{eq}^{mdh} = 10^{-4}$ mM [43]

$K_m^{mal} = 0.77$ mM [44]

$K_m^{nad} = 0.06$ mM [45]

$K_m^{oaa} = 0.04$ mM [44]

$K_m^{nadh} = 0.044$ mM [45]

Stoichiometric matrix

$v_{glc} = v_{glcT} - v_{hk}$

$v_{glc6p} = v_{hk} - v_{gpi}$

$v_{fru6p} = v_{gpi} - v_{pfk1} - v_{pfk2} + v_{fbp1}$

$v_{fru16p} = v_{pfk1} - v_{fbp1} - v_{ald}$

$v_{fru26p} = v_{pfk2} - v_{fru26bp}$

$v_{dhaP} = v_{ald} - v_{tpi}$

$v_{grap} = v_{ald} - v_{gapdh} + v_{tpi}$

$v_{bpg13} = -v_{pgk} + v_{gapdh}$

$v_{pg3} = v_{pgk} - v_{pgm}$

$v_{pg2} = v_{pgm} - v_{eno}$

$v_{pep} = v_{eno} - v_{pk}$

$v_{pyr} = v_{pk} - v_{ldh}$

$v_{atp} = v_{adk} - v_{hk} - v_{pfk1} - v_{pfk2} + v_{pgk} + v_{pk} + v_{ck}$

$v_{adp} = -2 \cdot v_{adk} + v_{hk} + v_{pfk1} + v_{pfk2} - v_{pgk} - v_{pk} - v_{ck}$

$v_{amp} = v_{adk}$

$v_{nadh} = v_{gapdh} - v_{ldh} + v_{mdh}$

$v_{nad} = -v_{nadh}$

$v_{oaa} = v_{mdh}$

$v_{mal} = -v_{mdh}$

$v_{p} = v_{fru26bp} - v_{gapdh} - v_{fbp1}$

$v_{cr} = v_{ck}$

$v_{crp} = -v_{ck}$

External concentrations of glucose, pyruvate, and lactate are considered to be constant:

$v_{glc_{ext}} = 0$

$v_{pyr_{ext}} = 0$

$v_{lac_{ext}} = 0$

Citric Acid Cycle and Redox Shuttle Systems

Rate equations for enzymes and transporters are given by:

Pyruvate transporter (mitochondrial membrane)

($pyr + h_{mito} \leftrightarrow pyr_{mito} + h$)

$$v_{pyrT} = v_{max}^{pyrT} \cdot \frac{\left(pyr - pyr_{mito} \cdot \frac{h_{mito}}{h_{cyt}}\right)}{\left(1 + \frac{pyr}{K_m^{pyr}}\right) + \left(1 + \frac{pyr_{mito}}{K_m^{Pyr_m}}\right) - 1}$$

$v_{max}^{pyrT} = 2 \cdot 10^3 \ \mu mol/g/s$

$K_m^{Pyr_m} = 0.15$ mM [46]

$K_m^{pyr} = 0.15$ mM [46]

Pyruvate dehydrogenase complex

($pyr_{mito} + coa + fad^{pdh} \leftrightarrow acoa + co_2 + fadh_2^{pdh}$)

($fadh_2^{pdh} + nad \leftrightarrow fad^{pdh} + nadh$)

$$v_{pdhc} = v_{max}^{pdhc} \frac{pyr_{mito}}{pyr_{mito} + K_m^{pyr}} \cdot \frac{fad^{pdh}}{fad^{pdh} + K_m^{fad^{pdh}}} \cdot \frac{coa_{mito}}{coa_{mito} + K_m^{coa_{mito}} \cdot \left(1 + \frac{acoa_{mito}}{K_i^{acoa_{mito}}}\right)} \cdot \left(1 + f_{ca} \frac{ca_{mito}}{ca_{mito} + K_a^{ca_{mito}}}\right)$$

$v_{max}^{pdh} = 8.24 \ \mu mol/g/s$

$K_M^{Pyr} = 0.068$ mM [47]

$K_m^{fad^{pdh}} = 10^{-5}$ mM

$K_m^{coa_{mito}} = 0.0047$ mM [48]

$K_i^{acao_{mito}} = 10^{-4}$ mM [48]

$f_{ca} = 1.7$ [49]

$K_a^{ca_{mito}} = 10^{-3}$ mM [50]

$$v_{pdhc-fad} = v_{max}^{pdhc-nad} \cdot \frac{fad^{pdh} \cdot nad_{mito} - \frac{1}{K_{eq}^{pdh^{fad}}} fad^{pdh} \cdot nadh_{mito}}{1 + \frac{nad_{mito}}{K_m^{nad_{mito}}}}$$

$v_{max}^{pdhc-fad} = 10^5$ μ$mol/g/s$

$K_m^{nad_{mito}} = 0.041$ mM [48]

$$K_{eq}^{pdh^{fad}} = exp\left(\frac{\left(n \cdot E_0^{nadh/nad} + n \cdot E_0^{FAD/FADH_2}\right) \cdot F}{R \cdot T}\right)$$

$E_0^{FAD/FADH_2} = -297mV$ [51]

$E_0^{nadh/nad} = -287mV$ [51]

$n = 2$

Citrate synthetase

(oaa + acoa ↔ cit)

$$v_{cs} = v_{max}^{cs} \cdot \frac{oaa_{mito}}{\left(oaa_{mito} + K_m^{oaa_{mito}} \cdot \left(1 + \frac{cit_{mito}}{K_i^{cit_{mito}}}\right)\right)} \cdot \frac{acoa_{mito}}{\left(acoa_{mito} + K_M^{acoa_{mito}} \cdot \left(1 + \frac{coa_{mito}}{K_i^{coa_{mito}}}\right)\right)}$$

$v_{max}^{cs} = 2.4 \cdot 10^3$ μ$mol/g/s$

$K_m^{oaa_{mito}} = 0.0045$ mM [52]

$K_i^{cit_{mito}} = 3.7$ mM [52]

$K_M^{acoa_{mito}} = 0.005$ mM [52]

$K_i^{coa_{mito}} = 0.025$ mM [52]

Aconitase

(cit ↔ isocit)

$$v_{ac} = v_{max}^{ac} \cdot \frac{cit_{mito} - \frac{1}{K_{eq}^{ac}} isocit_{mito}}{1 + \frac{cit_{mito}}{K_m^{cit}} + \frac{isocit_{mito}}{K_m^{isocit}}}$$

$v_{max}^{ac} = 10^6\ \mu mol/g/s$

$K_{eq}^{ac} = 0.067$ mM [53]

$K_m^{cit} = 0.48$ mM [54]

$K_m^{isocit} = 0.12$ mM [54]

Isocitrate dehydrogenase (NAD-dependent)

(isocit + nad ↔ akg + nadh)

$$v_{idh} = v_{max}^{idh} \cdot \frac{isocit_{mito}^n}{isocit_{mito}^n + \left(K_m^{isocit}\right)^n} \cdot \frac{nad_{mito}}{nad_{mito} + K_m^{nad_{mito}}}$$

$v_{max}^{idh} = 40\ \mu mol/g/s$

$n = 1.9$ [55]

$$K_m^{isocit} = \frac{K_{m1}^{isocit}}{1 + \left(\frac{ca_{mito}}{K_a^{ca}}\right)^{n_{ca}}} + K_{m2}^{isocit}$$

$K_{m1}^{isocit} = 0.11$ mM [56]

$K_{m2}^{isocit} = 0.06$ mM [56]

$K_a^{ca} = 0.0074$ mM [56]

$n_{ca} = 2$ [56]

$$K_m^{nad_{mito}} = K_0^{nad_{mito}} \cdot \left(1 + \frac{nadh_{mito}}{K_i^{nadh_{mito}}}\right)$$

$K_i^{nad_{mito}} = 0.091$ mM [55]

$K_0^{NADH} = 0.041$ mM [57]

Alpha-ketogluterate dehydrogenase

(akg + nad + coa ↔ succoa + nadh)

$$v_{kgdhc} = v_{max}^{kgdhc} \cdot \frac{akg_{mito}}{akg_{mito} + K_m^{akg_{mito}}} \cdot \frac{fad^{kgdhc}}{fad^{kgdhc} + K_m^{fad^{kgdhc}}} \cdot \frac{coa_{mito}}{coa_{mito} + K_m^{coa_{mito}} \cdot \left(1 + \frac{succoa_{mito}}{K_i^{succoa_{mito}}}\right)}$$

$v_{max}^{kgdhc} = 14\ \mu mol/g/s$

$K_m^{fad^{kgdhc}} = 10^{-5}$ mM

$K_m^{coa_{mito}} = 0.0013$ mM [58]

$K_i^{succoa_{mito}} = 0.0045$ mM [58]

$$K_m^{akg_{mito}} = \left(\frac{K_{m1}^{akg_{mito}}}{1 + \frac{ca_{mito}}{K_a^{ca_{mito}}}} + K_{m2}^{akg_{mito}}\right)\left(1 + \frac{nadh_{mito}}{K_i^{nadh_{mito}}}\right)$$

$K_{m1}^{akg_{mito}} = 2.5$ mM [59]

$K_{m2}^{akg_{mito}} = 0.13$ mM [59]

$K_a^{ca_{mito}} = 0.001$ mM [59]

$$v_{kgdhc}^{fad} = v_{max}^{kgdhc_{fad}} \cdot \frac{fadh_{kgdhc} \cdot nad_{mito} - \frac{1}{K_{eq}^{kgdhc_{fad}}} fad_{kgdhc} \cdot nadh_{mito}}{nadh_{mito} + K_m^{nad} \cdot \left(1 + \frac{nadh_{mito}}{K_i^{nadh_{mito}}}\right)}$$

$v_{max}^{kgdhc_{fad}} = 2 \cdot 10^7\ \mu mol/g/s$

$K_m^{nad} = 0.021$ mM [58]

$K_i^{nadh} = 0.0045$ mM [58]

$$K_{eq}^{kgdhc_{fad}} = \exp\left(\frac{\left(n \cdot E_0^{nadh/nad} + n\ \cdot E_0^{FAD/FADH_2}\right) \cdot F}{R \cdot T}\right)$$

$E_0^{FAD/FADH_2} = -297\ mV$ [51]

$E_0^{nadh/nad} = -287\ mV$ [51]

$n = 2$

Succinyl-CoA synthetase

(succoa + adp/gdp + p ↔ suc + coa + atp/gtp)

$$v_{scs} = v_{max}^{scs} \cdot \left(v_{scs}^{atp} + v_{scs}^{gtp}\right)$$

$$v_{max}^{scs} = v_0^{scs} \cdot \left(1 + f_p \cdot \frac{p_{mito}^n}{p_{mito}^n + \left(K_a^p\right)^n}\right)$$

$v_0^{scs} = 1.2 \cdot 10^4\ \mu mol/g/s$

$f_p = 1.2$ [60]

$n = 3$ [60]

$K_a^p = 2.5\ \ \mathrm{mM}$ [60]

$$v_{scs}^{atp} = \frac{succoa_{mito} \cdot adp_{mito} \cdot p_{mito} - \frac{1}{K_{eq}^{scs}} \cdot suc_{mito} \cdot coa_{mito} \cdot atp_{mito}}{\left(1 + \frac{succoa_{mito}}{K_m^{succoa}}\right)\left(1 + \frac{adp_{mito}}{K_m^{adp}}\right)\left(1 + \frac{p_{mito}}{K_m^p}\right) + \left(1 + \frac{suc_{mito}}{K_m^{suc}}\right)\left(1 + \frac{coa_{mito}}{K_m^{coa}}\right)\left(1 + \frac{atp_{mito}}{K_m^{atp_{mito}}}\right) - 1}$$

$K_{eq}^{scs} = 3.8\ \mathrm{mM}$ [61]

$K_m^{succoa} = 0.041\ \mathrm{mM}$ [62]

$K_m^{adp} = 0.25\ \mathrm{mM}$ [62]

$K_m^p = 0.72\ \mathrm{mM}$ [62]

$K_m^{suc} = 1.6\ \mathrm{mM}$ [63]

$K_m^{coa} = 0.056\ \mathrm{mM}$ [63]

$K_m^{atp_{mito}} = 0.017\ \mathrm{mM}$ [63]

$$v_{scs}^{gtp} = \frac{succoa_{mito} \cdot gdp_{mito} \cdot p_{mito} - \frac{1}{K_{eq}^{scs}} \cdot suc_{mito} \cdot coa_{mito} \cdot gtp_{mito}}{\left(1 + \frac{succoa_{mito}}{K_m^{succoa}}\right)\left(1 + \frac{gdp_{mito}}{K_m^{adp}}\right)\left(1 + \frac{p_{mito}}{K_m^p}\right) + \left(1 + \frac{suc_{mito}}{K_m^{suc}}\right)\left(1 + \frac{coa_{mito}}{K_m^{coa}}\right)\left(1 + \frac{gtp_{mito}}{K_m^{atp_{mito}}}\right) - 1}$$

$K_{eq}^{scs} = 3.8\ \mathrm{mM}$ [61]

$K_m^{succcoa} = 0.086$ mM [62]

$K_m^{gdp} = 0.007$ mM [62]

$K_m^{p} = 2.26$ mM [62]

$K_m^{suc} = 0.49$ mM [62]

$K_m^{coa} = 0.036$ mM [62]

$K_m^{gtp} = 0.036$ mM [62]

Succinate dehydrogenase

(suc + q ↔ fum + qh_2)

$$v_{sdh} = v_{max}^{sdh} \cdot \frac{suc_{mito} \cdot q - \frac{1}{K_{eq}^{sdh}} \cdot fum_{mito} \cdot qh_2}{suc_{mito} + K_m^{suc} \cdot \left(1 + \frac{mal_{mito}}{K_i^{mal}}\right)}$$

$v_{max}^{sdh} = 10^5\ \mu mol/g/s$

$K_m^{suc} = 1.6$ mM [64]

$K_i^{mal} = 2.2$ mM [65]

Fumarase

(fum ↔ mal)

$$v_{fum} = v_{max}^{fum} \cdot \frac{fum_{mito} - \frac{1}{K_{eq}^{fum}} mal_{mito}}{1 + \frac{fum_{mito}}{K_m^{fum}} + \frac{mal_{mito}}{K_m^{mal}}}$$

$v_{max}^{fum} = 4 \cdot 10^7\ \mu mol/g/s$

$K_{eq}^{fum} = 4.4$ mM [66]

$K_m^{fum} = 0.14$ mM [66]

$K_m^{mal} = 0.3$ mM [66]

Malate dehydrogenase (mitochondrial)

(mal + nad ↔ oaa + nadh)

$$v_{mdh_{mito}} = v_{max}^{mdh_{mito}} \cdot \frac{mal_{mito} \cdot nadh_{mito} - \frac{oaa_{mito} \cdot nadh_{mito}}{K_{eq}^{mdh_{mito}}}}{\left(1 + \frac{mal_{mito}}{K_m^{mal_{mito}}}\right)\left(1 + \frac{nad_{mito}}{K_m^{nad_{mito}}}\right) + \left(1 + \frac{oaa_{mito}}{K_m^{oaa_{mito}}}\right)\left(1 + \frac{nadh_{mito}}{K_m^{nadh_{mito}}}\right) - 1}$$

$v_{max}^{mdh_{mito}} = 2 \cdot 10^4\ \mu mol/g/s$

$K_{eq}^{mdh_{mito}} = 10^{-4}$ mM [67]

$K_m^{mal_{mito}} = 0.145$ mM [45]

$K_m^{nad_{mito}} = 0.06$ mM [45]

$K_m^{oaa_{mito}} = 0.017$ mM [45]

$K_m^{nadh_{mito}} = 0.044$ mM [45]

Nucleoside diphosphokinase (mitochondrial)

(atp + gdp ↔ adp + gtp)

$$v_{ndk_{mito}} = V_{max}^{ndk_{mito}} \cdot \left(\frac{atp_{mito} \cdot gdp_{mito} - {}^{1}\!/\!{}_{K_{eq}^{ndk}} \cdot adp_{mito} \cdot gtp_{mito}}{\left(1 + \frac{atp_{mito}}{K_m^{atp_{mito}}}\right) \cdot \left(1 + \frac{gdp_{mito}}{K_m^{gdp_{mito}}}\right) + \left(1 + \frac{adp_{mito}}{K_m^{adp_{mito}}}\right) \cdot \left(1 + \frac{gtp_{mito}}{K_m^{gtp_{mito}}}\right) - 1} \right)$$

$V_{max}^{ndk_{mito}} = 2 \cdot 10^3\ \mu mol/g/s$

$K_{eq}^{ndk} = 1$ [68]

$K_m^{atp_{mito}} = 1.66$ mM [69]

$K_m^{gdp_{mito}} = 0.036$ mM [69]

$K_m^{adp_{mito}} = 0.073$ mM [69]

$K_m^{gtp_{mito}} = 0.15$ mM [70]

Aspartate amino transferase

(asp + akg ↔ oaa + glu)

$$v_{aat} = v_{max}^{aat} \cdot \left(asp \cdot akg - \frac{1}{K_{eq}^{aat}} oaa \cdot glu \right)$$

$v_{max}^{aat} = 2 \cdot 10^3 \;\; \mu mol/g/s$

$K_{eq}^{aat} = 0.147$ mM [71]

Aspartate amino transferase (mitochondial)

(asp_{mito} + akg_{mito} ↔ oaa_{mito} + glu_{mito})

$$v_{aat_{mito}} = v_{max}^{aat_{mito}} \cdot \left(asp_{mito} \cdot akg_{mito} - \frac{1}{K_{eq}^{aat}} oaa_{mito} \cdot glu_{mito} \right)$$

$v_{max}^{aat_{mito}} = 2 \cdot 10^3 \; \mu mol/g/s$

$K_{eq}^{aat} = 0.147$ mM [71]

Aspartate-glutamate transporter

(asp_{mito} + glu + h ↔ asp + glu_{mito} + h_{mito})

$$v_{agT} = v_{max}^{agT} \cdot \frac{asp_{mito} \cdot glu_{cyt} - \frac{1}{K_{eq}^{agT}} asp_{cyt} \cdot glu_{mito}}{\left(1 + \frac{asp_{mito}}{K_m^{asp_{mito}}}\right) \cdot \left(\frac{glu_{cyt}}{K_m^{glu_{cyt}}}\right) + \left(1 + \frac{asp_{cyt}}{K_m^{asp_{cyt}}}\right) \cdot \left(1 + \frac{glu_{mito}}{K_m^{glu_{mito}}}\right) - 1}$$

$$K_{eq}^{agT} = exp\left(-v_{mm} \cdot \frac{F}{1000RT} + log\left(\frac{h_{cyt}}{h_{mito}}\right)\right)$$

$v_{max}^{AGT} = 4 \cdot 10^4 \; \mu mol/g/s$

$K_m^{asp_{mito}} = 0.05$ mM [72]

$K_m^{glu_{cyt}} = 2.8$ mM [72]

$K_m^{asp_{cyt}} = 0.05$ mM [72]

$K_m^{glu_{mito}} = 2.8$ mM [72]

Malate-α-ketoglutarate transporter

(mal + akg_{mito} ↔ mal_{mito} + akg)

$$v_{makgT} = v_{max}^{makgT} \cdot \frac{mal_{cyt} \cdot akg_{mito} - mal_{mito} \cdot akg_{cyt}}{\left(1 + \frac{mal_{cyt}}{K_m^{mal_{cyt}}}\right)\left(1 + \frac{akg_{mito}}{K_m^{akg_{mito}}}\right) + \left(1 + \frac{mal_{mito}}{K_m^{mal_{mito}}} + \right)\left(1 + \frac{akg_{cyt}}{K_m^{akg}}\right) - 1}$$

$v_{max}^{makT} = 2 \cdot 10^3\ \mu mol/g/s$

$K_m^{mal} = 1.36$ mM [73]

$K_M^{akg_{mito}} = 0.1$ mM [73]

$K_m^{mal_{mito}} = 0.71$ mM [73]

$K_m^{akg} = 0.2$ mM [73]

Glycerol-3-phosphate dehydrogenase (cytosolic)

(dhap + nadh ↔ nad + g3p)

$$v_{g3pdh} = v_{max}^{g3pdh} \cdot \frac{dhap \cdot nadh - \frac{1}{K_{eq}^{g3pdhc}} g3p \cdot nad}{\left(1 + \frac{dhap}{K_m^{dhap}}\right)\left(1 + \frac{nadh}{K_m^{nadh}}\right) + \left(1 + \frac{g3p}{K_m^{g3p}}\right)\left(1 + \frac{nad}{K_m^{nad}}\right) - 1}$$

$v_{max}^{g3pdh} = 4 \cdot 10^3\ \mu mol/g/s$

$K_{eq}^{g3pdhc} = 3257$ mM [74]

$K_m^{dhap} = 0.17$ mM [75]

$K_m^{nadh} = 0.01$ mM [75]

$K_m^{g3p} = 0.3$ mM [75]

$K_m^{nad} = 0.03$ [75]

Glycerol-3-phosphate dehydrogenase (mitochondrial)

(g3p + fad^{g3p} ↔ dhap +$fadh_2^{g3p}$)

($fadh_2^{g3p}$ + q ↔ fad^{g3p} + qh_2)

$$v_{g3pdh_{mito}} = v_{max}^{g3pdh} \cdot \frac{g3p \cdot fad^{g3pdh} - \frac{1}{K_{eq}^{g3pdh}} dhap \cdot fadh^{g3pdh}}{\left(1 + \frac{dhap}{K_m^{dhap}}\right) + \left(1 + \frac{g3p}{K_m^{g3p}}\right) - 1}$$

$$v_{max}^{g3pdh} = 10^5 \ \mu mol/g/s$$

$$K_{eq}^{g3pdh} = \exp\left(\frac{E_0 \cdot F}{R \cdot T}\right)$$

$$E_0 = -40mV$$

$K_m^{dhap} = 0.17 \text{ mM}$ [75]

$K_m^{g3p} = 0.3 \text{ mM}$ [75]

$$v_{g3pdh_{mito}-fad} = v_{max}^{g3pdh_{mito}-fad} \cdot q \cdot fadh^{g3pdh} - \frac{1}{K_{eq}^{g3pdh_{fad}}} qh_2 \cdot fad^{g3pdh}$$

$$v_{max}^{g3pdh_{mito}-fad} = 2 \cdot 10^6 \ \mu mol/g/s$$

$$K_{eq}^{g3pdh_{fad}} = \exp\left(\frac{\left(n \cdot E_0^{Q/QH_2} + n \cdot E_0^{FAD/FADH_2}\right) \cdot F}{R \cdot T}\right)$$

$E_0^{fad/fadh_2} = -210mV$ [76]

$E_0^{nadh/nad} = 190mV$ [76]

$$n = 2$$

Stoichiometric matrix

$$v_{glu} = v_{aat} - v_{agT} \cdot \frac{Vol_{mito}}{Vol_{axon}}$$

$$v_{glu_{mito}} = v_{aat_{mito}} - v_{agT}$$

$$v_{Pyr} = -v_{pyrT}$$

$$v_{Pyr_{mito}} = v_{pyrT} + v_{pdh}$$

$$v_{acoa_{mito}} = v_{pdh} - v_{cs}$$

$$v_{coa_{mito}} = -v_{kgdhc} + v_{scs} - v_{pdh} + v_{cs}$$

$$v_{oaa} = v_{aat}$$

$$v_{oaa_{mito}} = v_{mdh_{mito}} - v_{cs} + v_{aat_{mito}}$$

$$v_{mal} = -v_{makgT} \cdot \frac{Vol_{mito}}{Vol_{axon}}$$

$$v_{mal_{mito}} = v_{fum} + v_{makgT} - v_{mdh_{mito}}$$

$$v_{asp} = -v_{aat} + v_{agT} \cdot \frac{Vol_{mito}}{Vol_{axon}}$$

$$v_{asp_{mito}} = -v_{aat_{mito}} - v_{agT}$$

$$v_{akg} = -v_{aat} + v_{makgT} \cdot \frac{Vol_{mito}}{Vol_{axon}}$$

$$v_{akg_{mito}} = v_{idh} - v_{kgdhc} - v_{aat_{mito}} - v_{makgT}$$

$$v_{cit_{mito}} = v_{cs} - \mathrm{v_{ac}}$$

$$v_{isocit_{mito}} = \mathrm{v_{ac}} - v_{idh}$$

$$v_{succoa_{mito}} = v_{kgdhc} - v_{scs}$$

$$v_{suc_{mito}} = v_{scs} - v_{sdh}$$

$$v_{fum_{mito}} = v_{sdh} - v_{fum}$$

$$v_{atp_{mito}} = -v_{ndk_{mito}} + v_{scs}^{atp}$$

$$v_{adp_{mito}} = -v_{atp_{mito}}$$

$$v_{gtp_{mito}} = v_{ndk_{mito}} + v_{scs}^{gtp}$$

$$v_{gdp_{mito}} = -v_{gtp_{mito}}$$

$$v_{p_{mito}} = -v_{scs}^{atp} - v_{scs}^{gtp}$$

$$v_{nad_{mito}} = -v_{pdhc-fad} - v_{mdh_{mito}} - v_{kgdhc-fad} - v_{idh}$$

$$v_{nadh_{mito}} = -v_{nad_{mito}}$$

$$v_{nad} = v_{g3pdh}$$

$$v_{nadh} = -v_{g3pdh}$$

$$v_{dhap} = -v_{g3pdh_{mito}} + v_{g3pdh_{mito}-fad}$$

$$v_{g3p} = -v_{dhap}$$

$$v_{fad}^{pdhc} = -v_{pdhc} + v_{pdhc-fad}$$

$$v_{fadh}^{pdhc} = -v_{fad}^{pdhc}$$

$$v_{fad}^{kgdhc} = -v_{kgdhc} + v_{kgdhc-fad}$$

$$v_{fadh}^{kgdhc} = -v_{fad}^{kgdhc}$$

$$v_{fad}^{g3pdh_{mito}} = -v_{g3pdh_{mito}} + v_{g3pdh_{mito}-fad}$$

$$v_{fadh}^{g3pdh_{mito}} = -v_{fad}^{g3pdh_{mito}}$$

$$v_{fad}^{sdh} = -v_{sdh} + v_{sdh-fad}$$

$$v_{fadh}^{sdh} = -v_{fad}^{sdh}$$

$$v_q = -v_{sdh} \cdot \frac{Vol_{mito}}{Vol_{membrane}} - v_{g3pdh_{mito}-fad} \cdot \frac{Vol_{axon}}{Vol_{membrane}}$$

$$v_{qh_2} = -v_q$$

Respiratory Chain and Mitochondrial Electrophysiology

Ion currents in 10^{-4} A.

Rate equations for enzymes and transporters are given by:

Mitochondrial ATP exchanger [77]

(atp_{mito} + adp ↔ adp_{mito} + atp)

$$v_{ex} = v_{max}^{ex} \cdot n_m \cdot \frac{\left(1 - \frac{atp \cdot adp_{mito}}{adp \cdot atp_{mito}}\right) \cdot exp(U)}{\left(1 + \frac{atp}{adp} \cdot exp\left(S_{V_m} \cdot U\right)\right)\left(1 + \frac{adp_{mito}}{atp_{mito}}\right)}$$

$$v_{max}^{ex} = 1.08 \cdot 10^{-10} \ \cdot 10^{-4} A$$

$$S_{V_m} = 0.3$$

ATP synthetase

(atp ↔ adp + p)

$$v_{syn} = -v_{max}^{syn} \cdot n_m \cdot \left(adp_{mito} \cdot p_{mito} - \frac{1}{K_{eq}^{syn}} atp_{mito}\right)$$

$$v_{max}^{syn} = 1.5 \cdot 10^{-10} \ \cdot 10^{-4} A$$

$$K_{eq}^{syn} = exp\left(-\frac{dG_0^{syn}}{RT} - k \cdot U\right)\left(\frac{h_{cyt}}{h_m}\right)^k$$

$dG_0^{syn} = 30500$

$k = 3$ mM

Complex I

(nadh + q ↔ and + qh_2)

$$v_{cxI} = V_{max}^{cxI} \cdot \left(nadh_{mito} \cdot q_{mm} - {}^{1}/_{K_{eq}^{cxI}} \cdot nad_{mito} \cdot qh_{2_{mm}}\right)$$

$V_{max}^{cxI} = 0.0056 \cdot 10^{-4} A$

$$K_{eq}^{cxI} = \exp\left(\frac{\left(n \cdot E_0^{nadh/nad} + n \cdot E_0^{Q/QH_2} + n_H \cdot V_{mm}\right) \cdot F}{R \cdot T}\right) \cdot \left(\frac{h_{mito}}{h_{cyt}}\right)^{n_H}$$

$n = 2$

$E_0^{nadh/nad} = 297$ mV [51]

$E_0^{Q/QH_2} = 87 mV$ [76]

$n_H = 4$ [76]

Complex III

(qh_2 + $cytc_{ox}$ ↔ q + $cytc_{red}$)

$$v_{cxIII} = V_{max}^{cxIII} \cdot \left(qh2_{mm} \cdot cytc_{ox_{mm}} - {}^{1}/_{K_{eq}^{cxIII}} \cdot q_{mm} \cdot cytc_{red_{mm}}\right)$$

$V_{max}^{cxIII} = 11.25 \cdot 10^{-4} A$

$$K_{eq}^{cxIII} = \exp\left(\frac{\left(-n \cdot E_0^{Q/QH_2} + n \cdot E_0^{cytC_{ox}/cytC_{red}} + n \cdot v_{mm}\right) \cdot F}{R \cdot T}\right) \cdot \left(\frac{h_{mito}}{h_{cyt}}\right)^{n_{h_{mito}}}$$

$n = 2$

$E_0^{cytC_{ox}/cytC_{red}} = 221\, mV$ [78]

$E_0^{Q/QH_2} = 87\ mV$ [76]

$n_h = 4$

$h_{\emptyset} = 10^{-4} mM \stackrel{\text{def}}{=} \text{pH } 7$

Complex IV

(2 $cytc_{red}$ + o_2 + 4 h_{mito} ↔ h_2o + 2 $cytc_{ox}$ + 2 h)

$$v_{cxIV} = V_{max}^{cxIV} \cdot \left(\frac{cytc_{red}}{cytc_{red} + K_m^{cytc_{red}}}\right) \cdot \left(\frac{O_2}{O_2 + K_m^{O_2}}\right)$$

$V_{max}^{cxIV} = 1.92 \cdot 10^{-8} \cdot 10^{-4} A$

$K_m^{CytC_{red}} = 0.007$ mM [79]

$K_m^{O_2} = 3$ mmHG [80]

Oxygen diffusion

($o2_{ext}$ ↔ $o2_{axon}$)

$$v_{o_2}^{diff} = k_{o2}^{diff} \cdot (o_{2\,ext} - o_{2\,axon})$$

$k_{o2}^{diff} = 100\ \mu mol/mmHg/g/s$

Pump currents

Phosphate exchange, sodium, and potassium pumps as proton-driven antiport [81].

$$v_{Phos} = v_{max}^{Phos} \cdot A_m \cdot (p_{cyt} \cdot h_{cyt} - p_{mito} \cdot h_{mito})$$

$v_{max}^{Phos} = 100 \cdot 10^{-4} A$

$$I_{na-pump} = v_{max}^{na-pump} \cdot A_m \cdot (na_{cyt} \cdot h_{mito} - na_{mito} \cdot h_{cyt})$$

$v_{max}^{na-pump} = 13 \cdot 10^{-4} A$

$$I_{k-pump} = v_{max}^{k-pump} \cdot A_m \cdot (k_{cyt} \cdot h_{mito} - k_{mito} \cdot h_{cyt})$$

$v_{max}^{k-pump} = 9.75 \cdot 10^{-4} A$

Protons pumped by Complex I, III, and IV

$$I_{H_P} = -(4v_{C1} + 2v_{C3} + 4v_{C4})$$

Proton leak

$$I_{H_{ed}} = -2 \cdot A_m \cdot P_H \cdot U \cdot F \cdot \frac{h_{cyt} - h_{mito} \cdot \exp(U)}{1 - \exp(U)}$$

$$P_H = 2 \cdot 10^{-4} \frac{m}{s}$$

Sodium leak

$$I_{Na_{ed}} = -A_m \cdot P_{na} \cdot U \cdot F \cdot \frac{na_{cyt} - na_{mito} \cdot \exp(U)}{1 - \exp(U)}$$

$$P_{Na} = 0.1 \ \cdot 10^{-9} \frac{m}{s}$$

Potassium leak

$$I_{K_{ed}} = -2 \cdot A_m \cdot P_K \cdot U \cdot F \cdot \frac{k_{cyt} - k_{mito} \cdot \exp(U)}{1 - \exp(U)}$$

$$P_K = 0.2 \ \cdot 10^{-9} \frac{m}{s}$$

Chloride leak

$$I_{Cl_{ed}} = A_m \cdot P_{Cl} \cdot U \cdot F \cdot \frac{cl_{cyt} - cl_{mito} \cdot \exp(-U)}{1 - \exp(-U)}$$

$$P_{Cl} = 0.5 \ \cdot 10^{-9} \frac{m}{s}$$

Calcium–proton exchanger

$$I_{CaP_H} = -P_{CaP_H} \cdot \frac{ca_{mito}}{ca_{mito} + K_m^{ca_{mito}}} \cdot \left(ca_{mito} \cdot h_{cyt}^n - \frac{ca_{cyt} \cdot h_{mito}^n}{\exp(-U)} \right)$$

$$P_{CaP_H} = 2 \ \cdot 10^4$$

$K_m^{ca_{mito}} = 0.01$ mM [82]

$n = 3$

Calcium–sodium exchanger

$$I_{CaP_{Na}} = -P_{CaP_{Na}} \cdot n_m \cdot \frac{ca_{mito}}{ca_{mito} + K_m^{ca}} \cdot \frac{na_{cyt}^n}{na_{cyt}^n + (K_m^{na})^n} \cdot \left(ca_{mito} \cdot (na_{cyt})^{n_{na}} - \frac{ca_{cyt} \cdot (na_{mito})^{n_{Na}}}{K_{eq}^{ca-na}} \right)$$

$P_{CaP_{Na}} = 10^{-9} \frac{m}{s}$

$K_{eq}^{ca-na} = \exp(-U)$

$K_m^{na} = 8$ mM [81]

$K_m^{ca} = 0.0096$ mM [83]

$n_{Na} = 3$

$n = 2.8$ [81]

Calcium currents

$$I_{Ca_{ed}} = 2A_m \cdot U \cdot F \cdot \frac{ca_{cyt} - ca_{mito} \cdot \exp(2U)}{1 - \exp(2U)} \cdot \left(P_{Ca}^{rmc} \cdot \left(1 - \frac{ca_{cyt}}{ca_{cyt} + K_i^{ca}}\right) - P_{Ca}^{mcu} \cdot \frac{ca_{cyt}^n}{ca_{cyt}^n + (K_{m-Ca}^{mcu})^n} \cdot \frac{ca_{cyt}^{n_a}}{ca_{cyt}^{n_a} + (K_a^{ca})^{n_a}} \right)$$

$P_{Ca}^{rmc} = 0.6 \cdot 10^{-6} \ \frac{m}{s}$

$K_i^{Ca} = 0.0001$ mM [84]

$P_{ca}^{mcu} = 0.24 \cdot 10^{-2} \ \frac{m}{s}$

$K_{m-Ca}^{mcu} = 19.2$ mM [85]

$K_a^{ca} = 0.0005$ mM [85]

$n = 0.6$ [85]

$n_a = 5$ [85]

ATP-dependent cytosolic calcium increase

$$v_{ca-atp\ dependent} = k \cdot \left(ca_{\Delta} \cdot \left(atp_{ref} - atp\right) - ca_{cyt}\right)$$

$k = 10^4$ 1/s

$ca_{\Delta} = 0.0001$

$atp_{ref} = 3.5\ mM$

Stoichiometric matrix

$$v_{atp} = \frac{v_{ex}}{Vol_{axon}}$$

$$v_{adp} = -v_{atp}$$

$$v_{atp_{mito}} = -10^{-1} \cdot \frac{v_{ex}}{F \cdot Vol_{mito}} - 10^{-1} \cdot \frac{v_{syn}}{F \cdot Vol_{mito}}$$

$$v_{adp_{mito}} = -v_{atp_{mito}}$$

$$v_{p} = -10^{-1} \cdot \frac{v_{phos}}{F \cdot Vol_{axon}}$$

$$v_{p_{mito}} = 10^{-1} \cdot \frac{v_{phos} + v_{syn}}{F \cdot Vol_{mito}}$$

$$v_{nad_{mito}} = 10^{-1} \cdot \frac{v_{cxI}}{F \cdot Vol_{mito}}$$

$$v_{nadh_{mito}} = -v_{nad_{mito}}$$

$$v_{o_2} = -10^{-1} \cdot \frac{v_{cxIV}}{F \cdot Vol_{ax}} \cdot 0.25 + v_{o_2}^{diff}$$

$$v_{q} = 10^{-1} \cdot \frac{-v_{cxI} + v_{cxIII}}{F \cdot Vol_{membr}}$$

$$v_{qh_2} = -v_{q}$$

$$v_{cytc_{ox}} = 10^{-1} \cdot \frac{-2v_{cxIII} + v_{cxIV}}{F \cdot Vol_{membran}}$$

$$v_{cytc_{red}} = -v_{cytc_{ox}}$$

$$v_{h_{mito}} = 10^{-1} \cdot \frac{I_H}{F \cdot Vol_{mito}} \quad with$$

$$I_H = I_{H_{ed}} + I_{H_P} - I_{Na_P} - I_{K_P} + v_{phos} - 3 \cdot v_{syn} - 3 \cdot I_{CaP_H}$$

$$v_{na} = -10^{-1} \cdot \frac{I_{Na}}{F \cdot Vol_{axon}} \quad with\ I_{Na} = I_{Na_{ed}} + I_{Na_P} - 3 \cdot I_{CaP_{Na}}$$

$$v_{na_{mito}} = 10^{-1} \cdot \frac{I_{Na}}{F \cdot Vol_{mito}} \quad with\ I_{Na} = I_{Na_{ed}} + I_{Na_P} - 3 \cdot I_{CaP_{Na}}$$

$$v_K = -10^{-1} \cdot \frac{I_K}{F \cdot Vol_{axon}} \quad with\ I_K = I_{K_{ed}} + I_{K_P}$$

$$v_{K_m} = 10^{-1} \cdot \frac{I_K}{F \cdot Vol_{mito}} \quad with\ I_K = I_{K_{ed}} + I_{K_P}$$

$$v_{Cl} = -10^{-1} \cdot \frac{I_{Cl_{ed}}}{F \cdot Vol_{axon}}$$

$$v_{Cl_m} = 10^{-1} \cdot \frac{I_{Cl_{ed}}}{F \cdot Vol_{mito}}$$

$$v_{ca} = v_{ca-atp\ dependent}$$

$$v_{Ca_m} = 10^{-1} \cdot \frac{I_{Ca}}{F \cdot Vol_{mito}} \quad with\ I_{Ca} = I_{Ca_{ed}} + I_{CaP_H} + I_{CaP_{Na}}$$

$$v_{V_{mm}} = 10^{-1} \cdot \frac{-I_{Cl_{ed}} + I_K + I_H + I_{Na} - v_{phos} + v_{ex} + 2 \cdot I_{Ca}}{C_m \cdot A_m}$$

External conditions

All simulations were performed assuming constant external metabolic conditions. Following Berndt et al., 2015 [86] we used external glucose concentration of 2.4 mM [87, 88], external lactate of 1.5 mM [89, 90], and external oxygen of 60 mmHg [91].

As in Berndt et al., 2015 [86], total cytosolic adenine nucleotide pool was set to 3.4 mM, mitochondrial pool to 5 mM, cytosolic free nicotinamide adenine dinucleotide to 0.3 mM, mitochondrial free nicotinamide adenine dinucleotide to 0.05 mM, and total mitochondrial coenzyme A to 0.018 mM ([92, 93]; [94] and references within).

References

1. Hodgkin, A.L. and A.F. Huxley, *A quantitative description of membrane current and its application to conduction and excitation in nerve.* J Physiol, 1952. **117**(4): p. 500-44.
2. Kager, H., W.J. Wadman, and G.G. Somjen, *Conditions for the triggering of spreading depression studied with computer simulations.* J Neurophysiol, 2002. **88**(5): p. 2700-12.
3. Frankenhaeuser, B. and A.F. Huxley, *THE ACTION POTENTIAL IN THE MYELINATED NERVE FIBER OF XENOPUS LAEVIS AS COMPUTED ON THE BASIS OF VOLTAGE CLAMP DATA.* J Physiol, 1964. **171**: p. 302-15.
4. Berndt, N., S. Hoffmann, J. Benda, and H.G. Holzhutter, *The influence of the chloride currents on action potential firing and volume regulation of excitable cells studied by a kinetic model.* J Theor Biol, 2011. **276**(1): p. 42-9.
5. Larsen, B.R., A. Stoica, and N. MacAulay, *Managing Brain Extracellular K(+) during Neuronal Activity: The Physiological Role of the Na(+)/K(+)-ATPase Subunit Isoforms.* Front Physiol, 2016. **7**: p. 141.
6. Rushton, W.A., *A theory of the effects of fibre size in medullated nerve.* J Physiol, 1951. **115**(1): p. 101-22.
7. Huxley, A.F. and R. Stampfli, *Evidence for saltatory conduction in peripheral myelinated nerve fibres.* J Physiol, 1949. **108**(3): p. 315-39.
8. Waxman, J.D.K.a.P.K.S., *THE AXON, STRUCTURE, FUNCTION AND PATHOPHYSIOLOGY.* Oxford University Press, 1995.
9. Maher, F., T.M. DaviesHill, and I.A. Simpson, *Substrate specificity and kinetic parameters of GLUT3 in rat cerebellar granule neurons.* Biochemical Journal, 1996. **315**: p. 827-831.
10. Thompson, M.F. and Bachelar.Hs, *Cerebral-Cortex Hexokinase - Comparison of Properties of Solubilized Mitochondrial and Cytoplasmic Activities.* Biochemical Journal, 1970. **118**(1): p. 25-&.
11. Garfinkel, L., D. Garfinkel, P. Matsiras, and B. Matschinsky, *Kinetic-Properties of Hexokinase as Assembled with a Microcomputer Database.* Biochemical Journal, 1987. **244**(2): p. 351-357.
12. Tewari, Y.B., D.K. Steckler, and R.N. Goldberg, *Thermodynamics of isomerization reactions involving sugar phosphates.* J Biol Chem, 1988. **263**(8): p. 3664-9.
13. Gaitonde, M.K., E. Murray, and V.J. Cunningham, *Effect of 6-Phosphogluconate on Phosphoglucose Isomerase in Rat-Brain Invitro and Invivo.* Journal of Neurochemistry, 1989. **52**(5): p. 1348-1352.
14. Kasten, T.P., Y. Mhaskar, and G.A. Dunaway, *Regulation of Brain 6-Phosphofructo-1-Kinase - Effects of Aging, Fructose-2,6-Bisphosphate, and Regional Subunit Distribution.* Molecular and Cellular Biochemistry, 1993. **120**(1): p. 61-68.
15. Vora, S., R. Oskam, and G.E.J. Staal, *Isoenzymes of Phosphofructokinase in the Rat - Demonstration of the 3 Non-Identical Subunits by Biochemical, Immunochemical and Kinetic-Studies.* Biochemical Journal, 1985. **229**(2): p. 333-341.
16. Majumder, A.L. and F. Eisenberg, Jr., *Unequivocal demonstration of fructose-1,6-bisphosphatase in mammalian brain.* Proc Natl Acad Sci U S A, 1977. **74**(8): p. 3222-5.
17. Ventura, F., J.L. Rosa, S. Ambrosio, S.J. Pilkis, and R. Bartrons, *Bovine Brain 6-Phosphofructo-2-Kinase Fructose-2,6-Bisphosphatase - Evidence for a Neural-Specific Isozyme.* Journal of Biological Chemistry, 1992. **267**(25): p. 17939-17943.
18. Oakhill, J.S., R. Steel, Z.P. Chen, J.W. Scott, N. Ling, S. Tam, and B.E. Kemp, *AMPK Is a Direct Adenylate Charge-Regulated Protein Kinase.* Science, 2011. **332**(6036): p. 1433-1435.
19. Oakhill, J.S., Z.P. Chen, J.W. Scott, R. Steel, L.A. Castelli, N.M. Ling, S.L. Macaulay, and B.E. Kemp, *beta-Subunit myristoylation is the gatekeeper for initiating metabolic stress sensing by AMP-*

activated protein kinase (AMPK). Proceedings of the National Academy of Sciences of the United States of America, 2010. **107**(45): p. 19237-19241.
20. Veech, R.L., L. Raijman, K. Dalziel, and H.A. Krebs, *Disequilibrium in the triose phosphate isomerase system in rat liver.* Biochem J, 1969. **115**(4): p. 837-42.
21. Penhoet, E.E., M. Kochman, and W.J. Rutter, *Molecular and Catalytic Properties of Aldolase C.* Biochemistry, 1969. **8**(11): p. 4396-&.
22. Guix, F.X., G. Ill-Raga, R. Bravo, T. Nakaya, G. de Fabritiis, M. Coma, G.P. Miscione, J. Villa-Freixa, T. Suzuki, X. Fernandez-Busquets, M.A. Valverde, B. de Strooper, and F.J. Munoz, *Amyloid-dependent triosephosphate isomerase nitrotyrosination induces glycation and tau fibrillation.* Brain, 2009. **132**: p. 1335-1345.
23. Cori, C.F., S.F. Velick, and G.T. Cori, *The combination of diphosphopyridine nucleotide with glyceraldehyde phosphate dehydrogenase.* Biochim Biophys Acta, 1950. **4**(1-3): p. 160-9.
24. Kish, S.J., I. Lopes-Cendes, M. Guttman, Y. Furukawa, M. Pandolfo, G.A. Rouleau, B.M. Ross, M. Nance, L. Schut, L. Ang, and L. DiStefano, *Brain glyceraldehyde-3-phosphate dehydrogenase activity in human trinucleotide repeat disorders.* Archives of Neurology, 1998. **55**(10): p. 1299-1304.
25. Smith, C.M. and S.F. Velick, *The glyceraldehyde 3-phosphate dehydrogenases of liver and muscle. Cooperative interactions and conditions for functional reversibility.* J Biol Chem, 1972. **247**(1): p. 273-84.
26. Ryzlak, M.T. and R. Pietruszko, *Heterogeneity of Glyceraldehyde-3-Phosphate Dehydrogenase from Human-Brain.* Biochimica Et Biophysica Acta, 1988. **954**(3): p. 309-324.
27. Cornell, N.W., M. Leadbetter, and R.L. Veech, *Effects of free magnesium concentration and ionic strength on equilibrium constants for the glyceraldehyde phosphate dehydrogenase and phosphoglycerate kinase reactions.* J Biol Chem, 1979. **254**(14): p. 6522-7.
28. Sharma, H.K. and M. Rothstein, *Altered Brain Phosphoglycerate Kinase from Aging Rats.* Mechanisms of Ageing and Development, 1984. **25**(3): p. 285-296.
29. Rodwell, V.W., J.C. Towne, and S. Grisolia, *The kinetic properties of yeast and muscle phosphoglyceric acid mutase.* J Biol Chem, 1957. **228**(2): p. 875-90.
30. Ikura, K., H. Narita, R. Sasaki, and H. Chiba, *Immunochemical and Enzymatic Properties of "Bisphosphoglyceromutase-Phosphatase and Phosphoglyceromutase from Human Erythrocytes.* European Journal of Biochemistry, 1978. **89**(1): p. 23-31.
31. Batke, J., K.B. Nazaryan, and N.H. Karapetian, *Complex of Brain D-Phosphoglycerate Mutase and Gamma-Enolase and Its Reactivation by D-Glycerate 2,3-Bisphosphate.* Archives of Biochemistry and Biophysics, 1988. **264**(2): p. 510-518.
32. Schuster, R. and H.G. Holzhutter, *Use of mathematical models for predicting the metabolic effect of large-scale enzyme activity alterations. Application to enzyme deficiencies of red blood cells.* Eur J Biochem, 1995. **229**(2): p. 403-18.
33. Suzuki, F., Y. Umeda, and K. Kato, *Rat-Brain Enolase Isozymes - Purification of 3 Forms of Enolase.* Journal of Biochemistry, 1980. **87**(6): p. 1587-1594.
34. Schwark, W.S., R.L. Singhal, and G.M. Ling, *Metabolic Control Mechanisms in Mammalian Systems - Regulation of Pyruvate Kinase in Rat Cerebral Cortex.* Journal of Neurochemistry, 1971. **18**(1): p. 123-&.
35. Srivastava, L.K. and N.Z. Baquer, *Purification and Properties of Rat-Brain Pyruvate-Kinase.* Archives of Biochemistry and Biophysics, 1985. **236**(2): p. 703-713.
36. Williamson, D.H., P. Lund, and H.A. Krebs, *The redox state of free nicotinamide-adenine dinucleotide in the cytoplasm and mitochondria of rat liver.* Biochem J, 1967. **103**(2): p. 514-27.
37. Bonavita, V., G. Amore, and S. Avellone, *Molecular and Kinetic Properties of Lactate Dehydrogenase in Degenerating Peripheral Nerve.* Journal of the Neurological Sciences, 1966. **3**(4): p. 340-&.

38. Kocha, T., T. Fukuda, T. Isobe, and T. Okuyama, *Large-Scale Purification of Bovine Brain Lactate-Dehydrogenase by Affinity-Chromatography on Immobilized Colchicine.* Journal of Biochemistry, 1990. **107**(1): p. 138-143.
39. Nisselbaum, J.S., O. Bodansky, and D.E. Packer, *Comparison of Actions of Human Brain Liver + Heart Lactic Dehydrogenase Variants on Nucleotide Analogues + on Substrate Analogues in Absence + in Presence of Oxalate + Oxamate.* Journal of Biological Chemistry, 1964. **239**(9): p. 2830-&.
40. McKenna, M.C., J.T. Tildon, J.H. Stevenson, I.B. Hopkins, X.L. Huang, and R. Couto, *Lactate transport by cortical synaptosomes from adult rat brain: Characterization of kinetics and inhibitor specificity.* Developmental Neuroscience, 1998. **20**(4-5): p. 300-309.
41. Derr, R.F. and L. Zieve, *Adenylate energy charge: relation to guanylate energy charge and the adenylate kinase equilibrium constant.* Biochem Biophys Res Commun, 1972. **49**(6): p. 1385-90.
42. Sahlin, K., R.C. Harris, and E. Hultman, *Creatine-Kinase Equilibrium and Lactate Content Compared with Muscle Ph in Tissue Samples Obtained after Isometric-Exercise.* Biochemical Journal, 1975. **152**(2): p. 173-180.
43. Mueggler, P.A. and R.G. Wolfe, *Malate-Dehydrogenase - Kinetic Studies of Substrate Activation of Supernatant Enzyme by L-Malate.* Biochemistry, 1978. **17**(22): p. 4615-4620.
44. Malik, P., M.C. Mckenna, and J.T. Tildon, *Regulation of Malate-Dehydrogenases from Neonatal, Adolescent, and Mature Rat-Brain.* Neurochemical Research, 1993. **18**(3): p. 247-257.
45. Gelpi, J.L., A. Dordal, J. Montserrat, A. Mazo, and A. Cortes, *Kinetic studies of the regulation of mitochondrial malate dehydrogenase by citrate.* Biochem J, 1992. **283 (Pt 1)**: p. 289-97.
46. Halestrap, A.P., *The mitochondrial pyruvate carrier. Kinetics and specificity for substrates and inhibitors.* Biochem J, 1975. **148**(1): p. 85-96.
47. Land, J.M. and J.B. Clark, *Effect of phenylpyruvate on pyruvate dehydrogenase activity in rat brain mitochondria.* Biochem J, 1973. **134**(2): p. 539-44.
48. Blass, J.P. and C.A. Lewis, *Kinetic properties of the partially purified pyruvate dehydrogenase complex of ox brain.* Biochem J, 1973. **131**(1): p. 31-7.
49. Huang, H.M., L. Toral-Barza, K.F. Sheu, and G.E. Gibson, *The role of cytosolic free calcium in the regulation of pyruvate dehydrogenase in synaptosomes.* Neurochem Res, 1994. **19**(1): p. 89-95.
50. Denton, R.M. and J.G. McCormack, *The role of calcium in the regulation of mitochondrial metabolism.* Biochem Soc Trans, 1980. **8**(3): p. 266-8.
51. Moxley, M.A., D.A. Beard, and J.N. Bazil, *A pH-dependent kinetic model of dihydrolipoamide dehydrogenase from multiple organisms.* Biophys J, 2014. **107**(12): p. 2993-3007.
52. Matsuoka, Y. and P.A. Srere, *Kinetic studies of citrate synthase from rat kidney and rat brain.* J Biol Chem, 1973. **248**(23): p. 8022-30.
53. Blair, J.M., *Magnesium and the aconitase equilibrium: determination of apparent stability constants of mangnesium substrate complexes from equilibrium data.* Eur J Biochem, 1969. **8**(2): p. 287-91.
54. Guarriero-Bobyleva, V., M.A. Volpi-Becchi, and A. Masini, *Parallel partial purification of cytoplasmic and mitochondrial aconitate hydratases from rat liver.* Eur J Biochem, 1973. **34**(3): p. 455-8.
55. Willson, V.J. and K.F. Tipton, *Purification and characterization of ox brain NAD+-dependent isocitrate dehydrogenase.* J Neurochem, 1979. **33**(6): p. 1239-47.
56. Rutter, G.A. and R.M. Denton, *Regulation of NAD+-linked isocitrate dehydrogenase and 2-oxoglutarate dehydrogenase by Ca2+ ions within toluene-permeabilized rat heart mitochondria. Interactions with regulation by adenine nucleotides and NADH/NAD+ ratios.* Biochem J, 1988. **252**(1): p. 181-9.
57. Willson, V.J. and K.F. Tipton, *Allosteric properties of ox brain nicotinamide--adenine dinucleotide dependent isocitrate dehydrogenase.* J Neurochem, 1980. **34**(4): p. 793-9.

58. Smith, C.M., J. Bryla, and Williams.Jr, *Regulation of Mitochondrial Alpha-Ketoglutarate Metabolism by Product Inhibition at Alpha-Ketoglutarate Dehydrogenase.* Journal of Biological Chemistry, 1974. **249**(5): p. 1497-1505.
59. McCormack, J.G. and R.M. Denton, *The effects of calcium ions and adenine nucleotides on the activity of pig heart 2-oxoglutarate dehydrogenase complex.* Biochem J, 1979. **180**(3): p. 533-44.
60. Phillips, D., A.M. Aponte, S.A. French, D.J. Chess, and R.S. Balaban, *Succinyl-CoA synthetase is a phosphate target for the activation of mitochondrial metabolism.* Biochemistry, 2009. **48**(30): p. 7140-9.
61. Lynn, R. and R.W. Guynn, *Equilibrium constants under physiological conditions for the reactions of succinyl coenzyme A synthetase and the hydrolysis of succinyl coenzyme A to coenzyme A and succinate.* J Biol Chem, 1978. **253**(8): p. 2546-53.
62. Johnson, J.D., W.W. Muhonen, and D.O. Lambeth, *Characterization of the ATP- and GTP-specific succinyl-CoA synthetases in pigeon. The enzymes incorporate the same alpha-subunit.* J Biol Chem, 1998. **273**(42): p. 27573-9.
63. Krivanek, J. and L. Novakova, *A novel effect of vanadium ions: inhibition of succinyl-CoA synthetase.* Gen Physiol Biophys, 1991. **10**(1): p. 71-82.
64. Quinlan, C.L., A.L. Orr, I.V. Perevoshchikova, J.R. Treberg, B.A. Ackrell, and M.D. Brand, *Mitochondrial complex II can generate reactive oxygen species at high rates in both the forward and reverse reactions.* J Biol Chem, 2012. **287**(32): p. 27255-64.
65. Vinogradov, A.D., A.B. Kotlyar, V.I. Burov, and Y.O. Belikova, *Regulation of succinate dehydrogenase and tautomerization of oxaloacetate.* Adv Enzyme Regul, 1989. **28**: p. 271-80.
66. Kobayashi, K., T. Yamanishi, and S. Tuboi, *Physicochemical, catalytic, and immunochemical properties of fumarases crystallized separately from mitochondrial and cytosolic fractions of rat liver.* J Biochem, 1981. **89**(6): p. 1923-31.
67. Raval, D.N. and R.G. Wolfe, *Malic Dehydrogenase .4. Ph Dependence of Kinetic Parameters.* Biochemistry, 1962. **1**(6): p. 1118-&.
68. Lynn, R. and R.W. Guynn, *Equilibrium-Constants under Physiological Conditions for Reactions of Succinyl Coenzyme-a Synthetase and Hydrolysis of Succinyl Coenzyme-a to Coenzyme-a and Succinate.* Journal of Biological Chemistry, 1978. **253**(8): p. 2546-2553.
69. Kimura, N. and N. Shimada, *Membrane-Associated Nucleoside Diphosphate Kinase from Rat-Liver - Purification, Characterization, and Comparison with Cytosolic Enzyme.* Journal of Biological Chemistry, 1988. **263**(10): p. 4647-4653.
70. Fukuchi, T., N. Shimada, N. Hanai, N. Ishikawa, K. Watanabe, and N. Kimura, *Recombinant rat nucleoside diphosphate kinase isoforms (alpha and beta): purification, properties and application to immunological detection of native isoforms in rat tissues.* Biochim Biophys Acta, 1994. **1205**(1): p. 113-22.
71. Kishore, N., Y.B. Tewari, and R.N. Goldberg, *An investigation of the equilibrium of the reaction {L-aspartate(aq) plus 2-oxoglutarate(aq) = oxaloacetate(aq) plus L-glutamate(aq)}.* Journal of Chemical Thermodynamics, 1998. **30**(11): p. 1373-1384.
72. Dierks, T. and R. Kramer, *Asymmetric Orientation of the Reconstituted Aspartate Glutamate Carrier from Mitochondria.* Biochimica Et Biophysica Acta, 1988. **937**(1): p. 112-126.
73. Indiveri, C., T. Dierks, R. Kramer, and F. Palmieri, *Reaction-Mechanism of the Reconstituted Oxoglutarate Carrier from Bovine Heart-Mitochondria.* European Journal of Biochemistry, 1991. **198**(2): p. 339-347.
74. Euler, H., E. Adler, and G. Günther, *Über die Komponenten der Dehydrasesysteme. XV. - Zur Kenntnis der Dehydrierung von a-Glycerin-phosphorsäure im Tierkörper*, in *Hoppe-Seyler´s Zeitschrift für physiologische Chemie*. 1937. p. 1.
75. McGinnis, J.F. and J. de Vellis, *Purification and characterization of rat brain glycerol phosphate dehydrogenase.* Biochim Biophys Acta, 1974. **364**(1): p. 17-27.
76. Brown, G.C. and M.D. Brand, *Proton/electron stoichiometry of mitochondrial complex I estimated from the equilibrium thermodynamic force ratio.* Biochem J, 1988. **252**(2): p. 473-9.

77. Bohnensack, R., *The role of the adenine nucleotide translocator in oxidative phosphorylation. A theoretical investigation on the basis of a comprehensive rate law of the translocator.* J Bioenerg Biomembr, 1982. **14**(1): p. 45-61.
78. Hinkle, P. and P. Mitchell, *Effect of membrane potential on equilibrium poise between cytochrome a and cytochrome c in rat liver mitochondria.* J Bioenerg, 1970. **1**(1): p. 45-60.
79. Napiwotzki, J. and B. Kadenbach, *Extramitochondrial ATP/ADP-ratios regulate cytochrome c oxidase activity via binding to the cytosolic domain of subunit IV.* Biol Chem, 1998. **379**(3): p. 335-9.
80. Kasischke, K.A., E.M. Lambert, B. Panepento, A. Sun, H.A. Gelbard, R.W. Burgess, T.H. Foster, and M. Nedergaard, *Two-photon NADH imaging exposes boundaries of oxygen diffusion in cortical vascular supply regions.* J Cereb Blood Flow Metab, 2011. **31**(1): p. 68-81.
81. Bernardi, P., *Mitochondrial transport of cations: Channels, exchangers, and permeability transition.* Physiological Reviews, 1999. **79**(4): p. 1127-1155.
82. Gunter, T.E. and D.R. Pfeiffer, *Mechanisms by which mitochondria transport calcium.* Am J Physiol, 1990. **258**(5 Pt 1): p. C755-86.
83. Hilgemann, D.W., S. Matsuoka, G.A. Nagel, and A. Collins, *Steady-state and dynamic properties of cardiac sodium-calcium exchange. Sodium-dependent inactivation.* J Gen Physiol, 1992. **100**(6): p. 905-32.
84. Gunter, T.E., L. Buntinas, G. Sparagna, R. Eliseev, and K. Gunter, *Mitochondrial calcium transport: mechanisms and functions.* Cell Calcium, 2000. **28**(5-6): p. 285-96.
85. Kirichok, Y., G. Krapivinsky, and D.E. Clapham, *The mitochondrial calcium uniporter is a highly selective ion channel.* Nature, 2004. **427**(6972): p. 360-4.
86. Berndt, N., O. Kann, and H.G. Holzhütter, *Physiology-based kinetic modeling of neuronal energy metabolism unravels the molecular basis of NAD(P)H fluorescence transients.* J Cereb Blood Flow Metab, 2015. **35**(9): p. 1494-506.
87. Silver, I.A. and M. Erecinska, *Extracellular glucose concentration in mammalian brain: continuous monitoring of changes during increased neuronal activity and upon limitation in oxygen supply in normo-, hypo-, and hyperglycemic animals.* J Neurosci, 1994. **14**(8): p. 5068-76.
88. Gruetter, R., E.J. Novotny, S.D. Boulware, D.L. Rothman, G.F. Mason, G.I. Shulman, R.G. Shulman, and W.V. Tamborlane, *Direct measurement of brain glucose concentrations in humans by 13C NMR spectroscopy.* Proc Natl Acad Sci U S A, 1992. **89**(3): p. 1109-12.
89. Boretius, S., R. Tammer, T. Michaelis, J. Brockmoller, and J. Frahm, *Halogenated volatile anesthetics alter brain metabolism as revealed by proton magnetic resonance spectroscopy of mice in vivo.* Neuroimage, 2013. **69**: p. 244-55.
90. Lundgaard, I., M.L. Lu, E. Yang, W. Peng, H. Mestre, E. Hitomi, R. Deane, and M. Nedergaard, *Glymphatic clearance controls state-dependent changes in brain lactate concentration.* J Cereb Blood Flow Metab, 2017. **37**(6): p. 2112-2124.
91. Vovenko, E., *Distribution of oxygen tension on the surface of arterioles, capillaries and venules of brain cortex and in tissue in normoxia: an experimental study on rats.* Pflugers Arch, 1999. **437**(4): p. 617-23.
92. Greiner, J.V. and T. Glonek, *Intracellular ATP Concentration and Implication for Cellular Evolution.* Biology (Basel), 2021. **10**(11).
93. Pathak, D., L.Y. Shields, B.A. Mendelsohn, D. Haddad, W. Lin, A.A. Gerencser, H. Kim, M.D. Brand, R.H. Edwards, and K. Nakamura, *The role of mitochondrially derived ATP in synaptic vesicle recycling.* J Biol Chem, 2015. **290**(37): p. 22325-36.
94. Berndt, N., S. Bulik, I. Wallach, T. Wunsch, M. Konig, M. Stockmann, D. Meierhofer, and H.G. Holzhutter, *HEPATOKIN1 is a biochemistry-based model of liver metabolism for applications in medicine and pharmacology.* Nat Commun, 2018. **9**(1): p. 2386.
