# Supplementary material for: Metabolic implications of axonal demyelination and its consequences for synchronized network activity: An in silico and in vitro study
Source: J Cereb Blood Flow Metab. 2023 Apr 26;43(9):1571–87. doi: 10.1177/0271678X231170746 (PMC10414014; doi:10.1177/0271678X231170746)
Supplement: sj-pdf-2-jcb-10.1177_0271678X231170746 - Supplemental material for Metabolic implications of axonal demyelination and its consequences for synchronized network activity: An in silico and in vitro study [file sj-pdf-2-jcb-10.1177_0271678X231170746.pdf]

Supplementary Figures

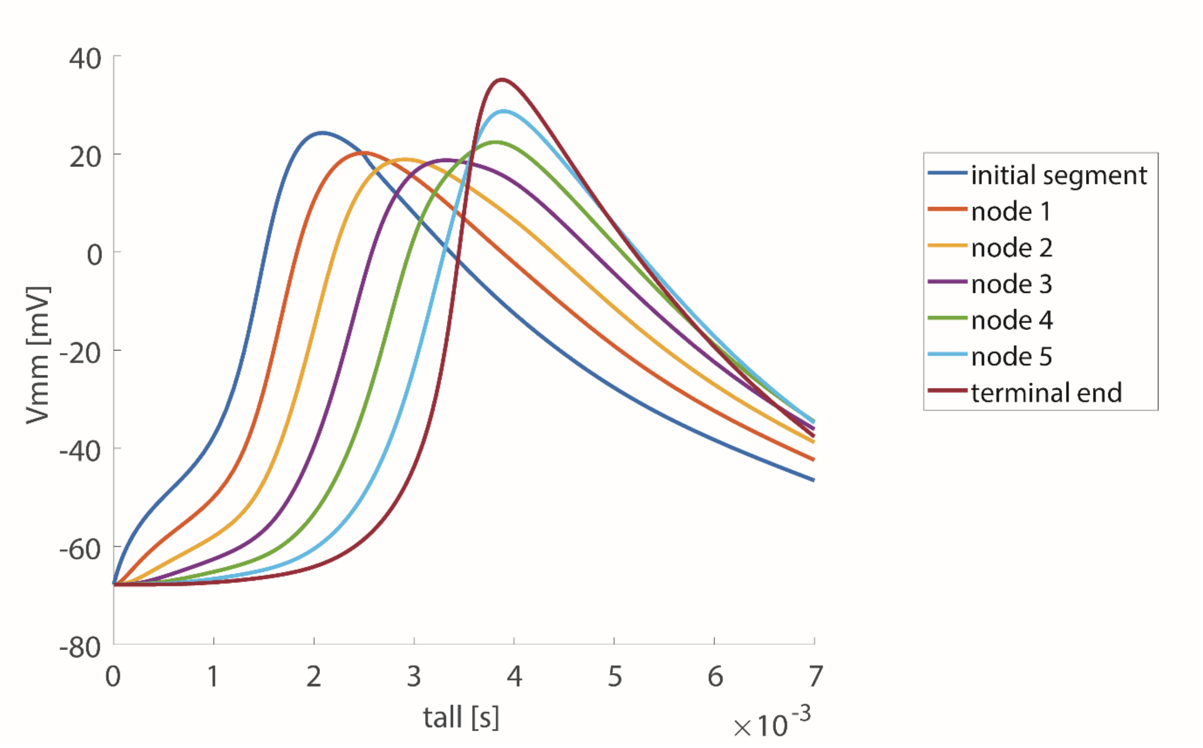

Fig. S1 Action potential (AP) propagation in a healthy axon. Maximal axonal membrane potential during AP propagation at the different nodes of Ranvier (NOR) is maintained.

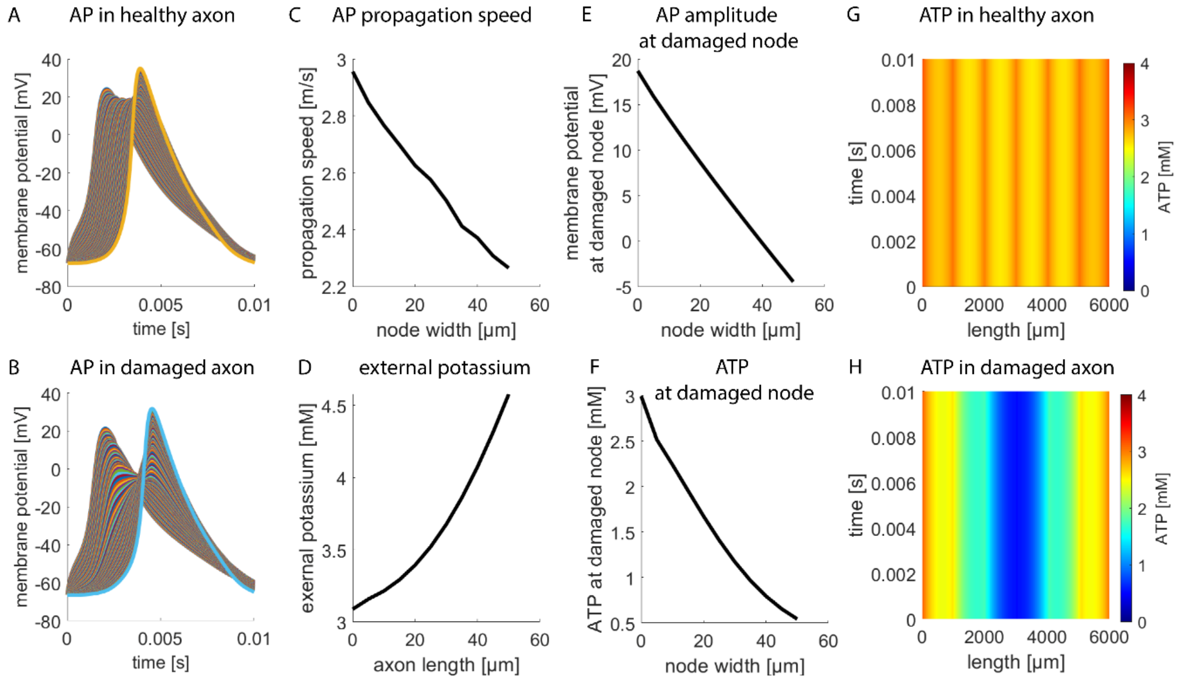

Fig. S2 Effects of node widening assuming an additional unspecific, high-affinity ATP consumer accounting for 30% of basal ATP production. **(A)** AP propagation over time in a healthy axon and **(B)** in an axon with 50 µm node widening at the 3rd NOR. The different curves give the membrane potential of each axon segment. The decreased AP amplitude results from the widening of the 3rd NOR. **(C)** AP propagation speed in dependence on node width. Propagation speed decreases in dependence on node widening at the 3rd NOR. **(D)** Extra-axonal potassium concentration around the damaged node in dependence on node widening. Increased node width leads to increasingly aberrant potassium concentrations around the damaged node. **(E)** Maximal axonal membrane potential during AP at the damaged node in dependence on node width. Increased node width decreases AP amplitude around the damaged node. **(F)** ATP concentration at the damaged node in dependence on node width. Increased node width leads to energy depletion at the damaged node. **(G)** Axonal ATP concentration in a healthy axon. The x-axis depicts the position along the length of the axon, y-axis depicts time along an AP (same as the x-axis in panels A-F). ATP concentrations are around 2.7 mM throughout the whole axon. Constancy over time indicated that perturbance by a single AP can be buffered by available ATP. **(H)** Axonal ATP concentration in an axon with 50 µm node widening at the 3rd NOR. ATP depletion centers around the damaged node but spreads through the axon.

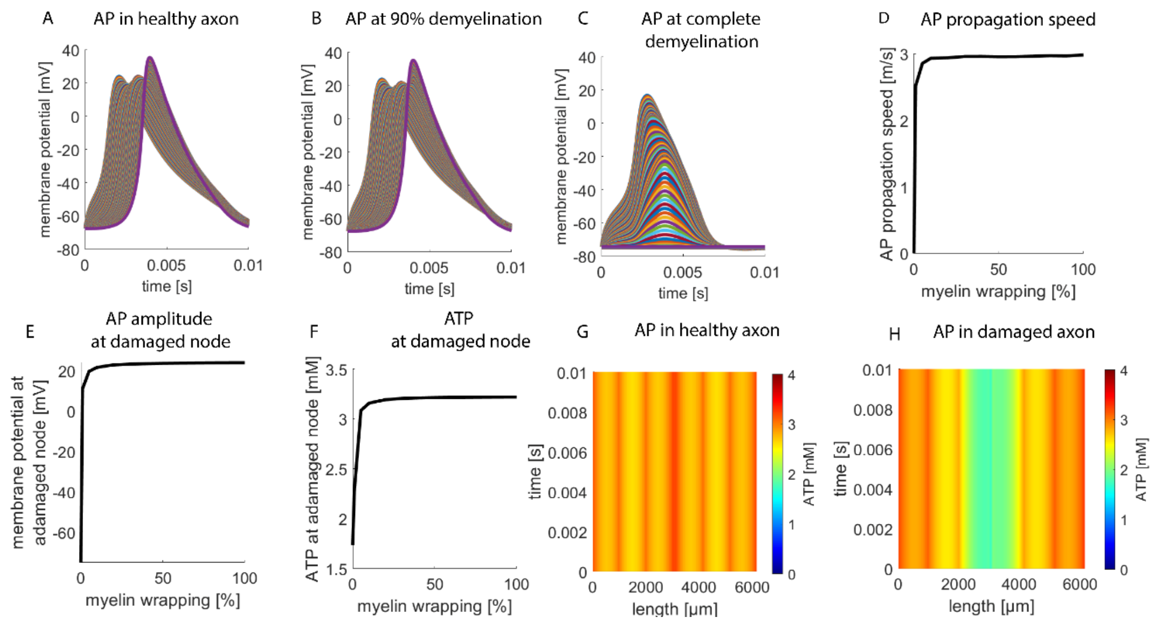

Fig. S3 Effects of paranodal demyelination widening assuming an additional unspecific, high-affinity ATP consumer accounting for 30% of basal ATP production. **(A)** AP propagation over time in a healthy axon, **(B)** in an axon with 90% paranodal demyelination, and **(C)** complete paranodal demyelination around the 3rd NOR. The different curves give the membrane potential of each axon segment. Complete paranodal demyelination leads to a breakdown of AP propagation. **(D)** AP propagation speed in dependence on the percentage of myelin wrappings around the paranode. At AP breakdown, propagation speed is zero. **(E)** Maximal axonal membrane potential during AP at the damaged node in dependence on the percentage of myelin wrappings around the paranode. At AP breakdown, there is no depolarization at the damaged node and subsequent axon segments. **(F)** ATP concentration at the damaged node in dependence on the percentage of myelin wrappings around the paranode. Complete paranodal demyelination leads to decreased ATP availability at the damaged node. **(G)** Axonal ATP concentration in a healthy axon. ATP concentrations are around 2.7 mM throughout the whole axon. **(H)** Axonal ATP concentration in an axon during complete paranodal demyelination at the 3rd NOR. Reduced ATP availability centers around the damaged node and affects adjacent axon segments.

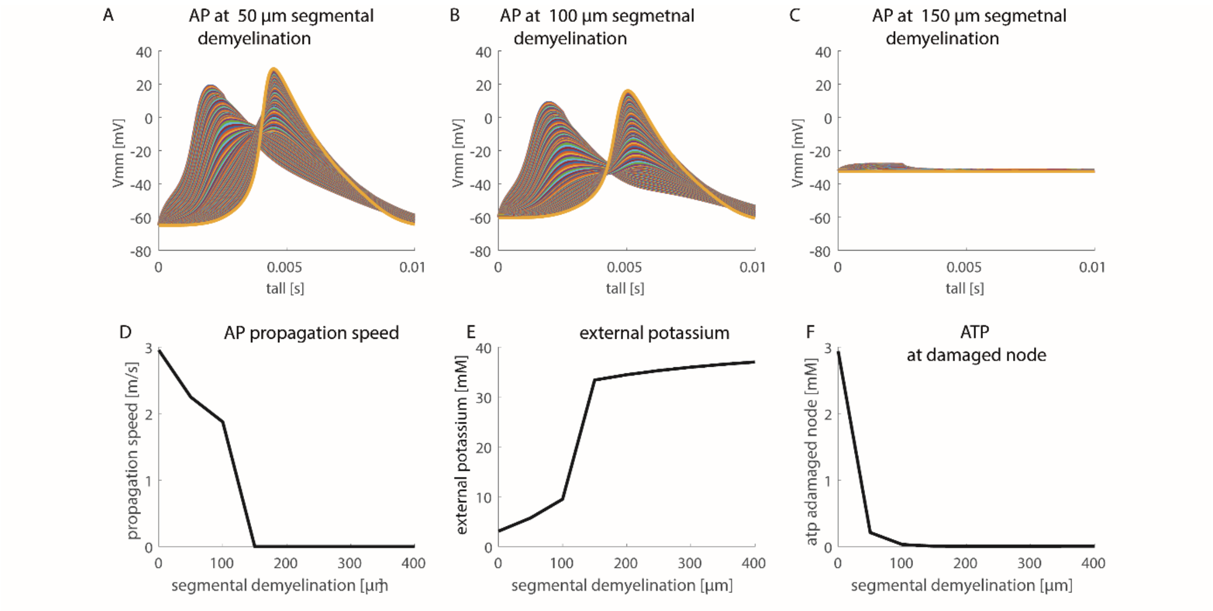

Fig. S4 Effects of segmental demyelination widening assuming an additional unspecific, high-affinity ATP consumer accounting for 30% of basal ATP production. Axonal membrane potential propagation at **(A)** 50 µm, **(B)** 100 µm, and **(C)** 150 µm segmental demyelination at the 3rd NOR. At 150 µm segmental demyelination, the whole axon depolarizes and AP breaks down. **(D)** AP propagation in dependence on the length of segmental demyelination. **(E)** Extra-axonal potassium concentration around the damaged node for different lengths of segmental demyelination. In dependence on the length of the demyelinated stretch, ion homeostasis is compromised. **(F)** ATP concentration at the damaged node in dependence on the length of segmental demyelination. Segmental demyelination leads to severe ATP depletion.

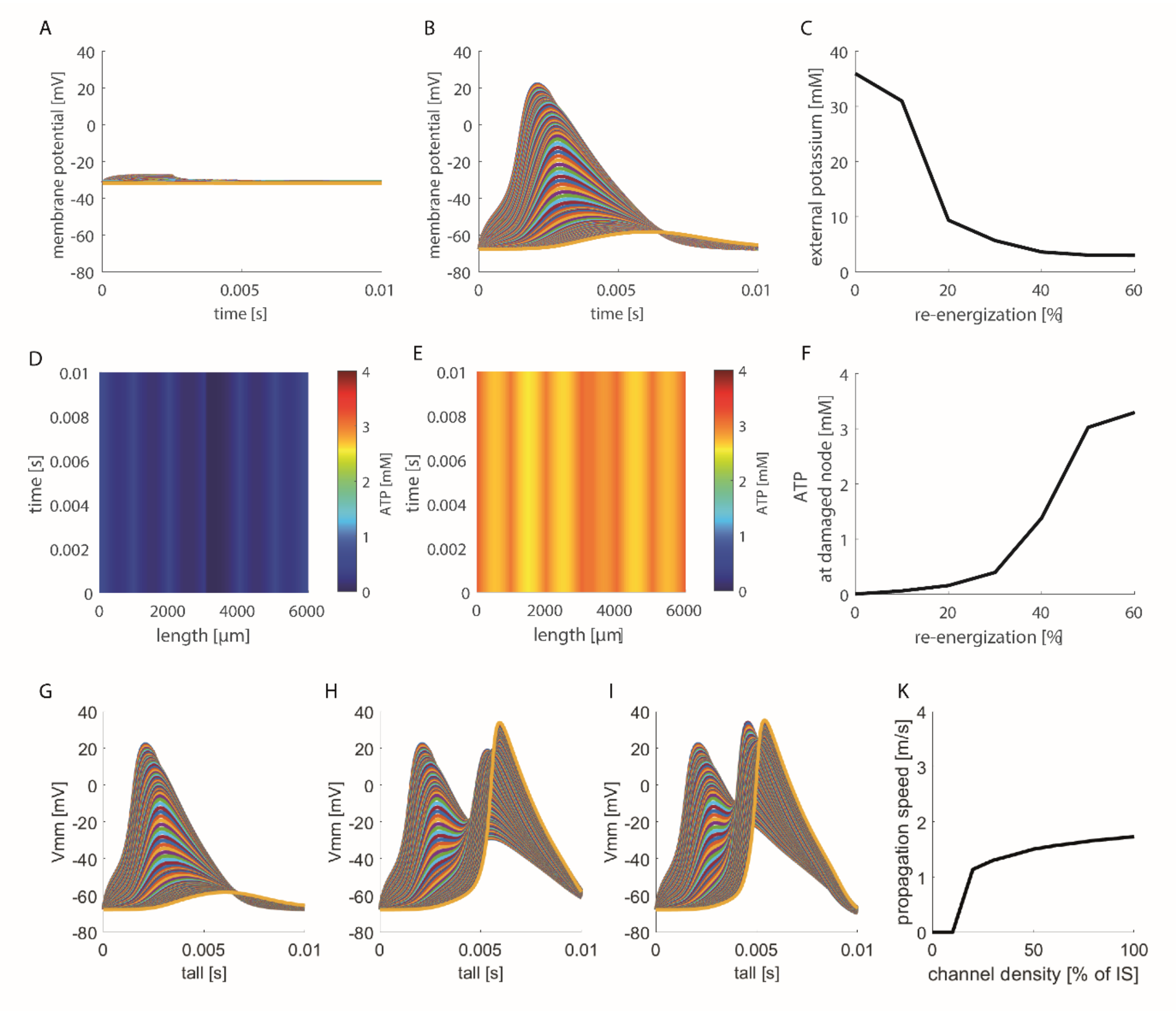

Fig. S5 Effect of re-energization and continuous propagation for segmentally demyelination axons assuming an additional unspecific, high-affinity ATP consumer accounting for 30% of basal ATP production. **(A)** Lack of AP without re-energization. **(B)** Re-energization corresponding to 50% of the ATP production capacity of the axon initial segment (AIS) leads to repolarization of the axonal membrane and AP initiation, but there is no AP propagation through the demyelinated membrane stretch. **(C)** Re-energization decreases extracellular potassium concentration. At re-energization corresponding to 50% of the ATP production capacity of the AIS, ion homeostasis is reestablished. **(D)** ATP concentration without re-energization. ATP is depleted throughout the axon. **(E)** With re-energization corresponding to 50% of the AIS, ATP availability is re-established through the whole axon. **(F)** ATP availability at the damaged node in dependence on re-energization in the percentage of AIS ATP production capacity. A protein density of 50% compared to the AIS reconstitutes ATP availability. **(G)** AP propagation without additional ion channels. APs can be elicited, but do not propagate through the demyelinated axon stretch. **(H)** With 50% AIS channel density and **(I)** 100% AIS channel density. APs propagate through the complete axon, although at different speeds. **(K)** AP propagation speed in dependence on the degree of channel density. AP propagation speed reaches saturation and cannot be increased by a higher channel density. The resulting AP propagation speed is significantly decreased compared to myelinated axons.
